# c-Rel drives pancreatic cancer metastasis through fibronectin-integrin signaling-induced isolation stress resistance and EMT

**DOI:** 10.1186/s12943-025-02486-5

**Published:** 2025-12-15

**Authors:** D. Bakırdöğen, K. Görgülü, J. Xin, L. Richter, S. Alcalá, L. Ruiz-Cañas, C. Dai, K. J. Frank, N. Wu, K. N. Diakopoulos, H. Ozturk, D. Demircioğlu, K. Peschke, R. Ranjan, F. Fusco, J. Martinez-Useros, M. J. Fernandez-Aceñero, N. F. Chhabra, J. C. López-Gil, J. Ai, D. A. Ruess, E. Kaya-Aksoy, F. Schmidt, L. Kohlmann, A. Berninger, H. Yang, F. Schicktanz, K. Steiger, I. E. Demir, R. M. Schmid, M. Reichert, M. Adli, M. Lesina, B. Sainz, H. Algül

**Affiliations:** 1 Comprehensive Cancer Center München, Institute for Tumor Metabolism, Klinikum rechts der Isar, School of Medicine and Health, Technical University of Munich, Ismaninger Str. 22, Munich, 81675 Germany; 2 Department of Surgery, Klinikum Rechts der Isar, Technical University of Munich, Munich, Germany; 3 Cancer Stem Cells and Fibroinflammatory Microenvironment Group, Cancer Department, Instituto de Investigaciones Biomédicas (IIBM) Sols-Morreale CSIC-UAM, Madrid, Spain; 4 Biomarkers and Personalized Approach to Cancer Group (BIOPAC), Area 3 Cancer, Instituto Ramón y Cajal de Investigación Sanitaria (IRYCIS), Madrid, 28049 Spain; 5 Centro de Investigación Biomédica en Red, Área Cáncer, CIBERONC, ISCIII, Madrid, Spain; 6 Department of Obstetrics and Gynecology, Robert Lurie Comprehensive Cancer Center, Feinberg School of Medicine at Northwestern University, Chicago, IL USA; 7 Department of Oncological Sciences, Icahn School of Medicine at Mount Sinai, New York, NY 10029 USA; 8 Tisch Cancer Institute, Icahn School of Medicine at Mount Sinai, New York, NY 10029 USA; 9 Bioinformatics for Next-Generation Sequencing (BiNGS) core, Icahn School of Medicine at Mount Sinai, New York, NY 10029 USA; 10 Translational Pancreatic Cancer Research Center, TUM School of Medicine and Health, Department of Clinical Medicine – Clinical Department for Internal Medicine II, University Medical Center, Technical University of Munich, Munich, Germany; 11 TUM School of Medicine and Health, Department of Clinical Medicine – Clinical Department for Internal Medicine II, University Medical Center, Technical University of Munich, Munich, Germany; 12 Center for Protein Assemblies (CPA), Technical University of Munich, Munich, Germany; 13 Center for Organoid Systems (COS), Technical University of Munich, Munich, Germany; 14 German Cancer Consortium (DKTK), partner site Munich, Munich, Germany; 15 Bavarian Cancer Research Center (BZKF), Munich, Germany; 16 Institute of Pathology, School of Medicine, Technical University of Munich, Munich, Germany; 17 Translational Oncology Division, Oncohealth Institute, Fundacion Jiménez Díaz University Hospital, Madrid, 28040 Spain; 18 Area of Physiology, Department of Basic Health Sciences, Faculty of Health Sciences, Rey Juan Carlos University, Madrid, 28922 Spain; 19 Pathology Department, Clinico San Carlos University Hospital, Madrid, 28040 Spain; 20 Department of Gastroenterology, The First Affiliated Hospital of Nanchang University, Nanchang, Jiangxi Province China; 21 Department of General and Visceral Surgery, Center for Surgery, Medical Center University of Freiburg, Freiburg, Germany; 22 German Cancer Consortium (DKTK), Partner Site Freiburg and German Cancer Research Center (DKFZ), Heidelberg, Germany

**Keywords:** PDAC, c-Rel, NF-κB, Integrin β3, CD61, Fibronectin, FN1, Metastasis, Anchorage independence

## Abstract

**Background:**

Pancreatic ductal adenocarcinoma (PDAC) remains one of the deadliest malignancies, with limited treatment options and a high recurrence rate. Recurrence often occurs with metastasis, for which cancer cells must adapt to isolation stress to successfully colonize distant organs. While the fibronectin–integrin axis has been implicated in this adaptation, its regulatory mechanisms require further elaboration.

**Methods:**

We utilized genetically engineered PDAC mouse models with c-Rel knockout, overexpression and fibronectin (FN1) depletion, alongside in vitro assays, to assess EMP, extracellular matrix (ECM) remodeling, and resistance to anchorage-independent growth. Functional analyses, including transcriptomics, Cut&Run, flow cytometry, immunohistochemistry, and metastatic assays, were performed to elucidate the role of c-Rel in fibronectin–integrin signaling during PDAC progression.

**Results:**

We identified c-Rel as an oncogenic driver in PDAC that promotes EMP, ECM remodeling, and survival under isolation stress. c-Rel directly regulates FN1 and CD61/integrin β3 (ITGB3) transcription, enhancing cellular adaptability in metastatic settings. While FN1 is dispensable for EMT, its absence significantly impairs metastatic colonization and anchorage-independent growth.

**Conclusions:**

Our findings suggest that c-Rel can regulate PDAC progression and metastasis by modulating the tumor microenvironment and stress resistance. Targeting the c-Rel–fibronectin–integrin axis may offer novel therapeutic strategies to mitigate disease progression.

**Supplementary Information:**

The online version contains supplementary material available at 10.1186/s12943-025-02486-5.

Pancreatic cancer (PDAC) is the third leading cause of cancer-related death, with a 13% 5-year survival rate in the United States [1]. Although the overall cancer mortality for all cancers has tended to decline, the incidence of pancreatic cancer has increased [1]. Projections for both 2030 and 2040 suggest that the number of deaths related to pancreatic cancer will be the second highest, trailing behind that related to lung cancer [2, 3]. Nevertheless, treatment options are still limited [4]. Patients with resected PDAC tumors recur rather quickly (~ 75%), partially owing to the presence of circulating tumor cells (CTCs) and the establishment of distant micrometastases (~ 60%) [5, 6]. Although adjuvant chemotherapy may reduce metastatic recurrence, more effective targeted approaches are needed.

Within the metastatic cascade, once in circulation, cancer cells adapt to overcome isolation stress [7]. Tolerance to isolation stress induced by limiting survival conditions, including nutrient stress, hypoxia, oxidative stress, or detachment from the extracellular matrix (ECM), can increase cancer cell resilience. These cancer cells can demonstrate increased stemness, tumor-initiating capacity, and metastasis. Recently, an enriched fibronectin–integrin axis was proposed to increase isolation stress tolerance in PDAC [8]. In parallel, multiple studies have identified various subclusters of pancreatic CTCs that express ECM-associated genes [9–12]. An expanded understanding of the signaling networks regulating the cellular niche under isolation stress is needed to develop novel therapies against metastatic PDAC recurrence.

In addition to its multiple components, NF-κB is a ubiquitous signaling pathway that is active in various physiological and pathophysiological processes [13]. Owing to technical limitations, NF-κB signaling has long been thought to exhibit binary activation (on/off). However, recent advances in techniques, enabling single-cell resolution for analysis, have revealed multiple quantitative features of NF-κB signaling dynamics [14]. Despite these technical advances, the functional roles of individual NF-κB components in pancreatic pathophysiology remain poorly understood. Among the five NF-κB transcription factors, RelA, RelB, and c-Rel contain a transactivation domain (TAD), whose heterodimers can induce the transcriptional activation of target genes. Previously, our group demonstrated a context-specific function of the canonical NF-κB transcription factor RelA (p65) in PDAC [15]. Interestingly, during carcinogenesis, RelA assumes a tumor suppressor role because of its involvement in senescence activation in cancer precursors. However, once the senescence checkpoint is exceeded, the oncogenic function of RelA becomes rather operative. Additionally, the absence of the noncanonical NF-κB transcription factor RelB has been shown to decelerate pancreatic carcinogenesis [16].

In contrast, the REL proto-oncogene NF-kB subunit (c-Rel) has not been studied extensively in solid tumors, including PDAC. Both tumor suppressor [17–19] and oncogenic [20–23] roles for c-Rel in cancer have been reported. Additional studies have highlighted the involvement of c-Rel in TNF-related apoptosis-inducing ligand (TRAIL) resistance [24], epithelial-to-mesenchymal transition (EMT) [25–28], cancer stem cell characteristics (CSCness) [28, 29], and immunomodulatory effects [30–33] in various cancers.

In this study, via the use of genetically engineered mouse models (GEMMs) of PDAC, we revealed a predominantly oncogenic role for increased c-Rel protein activity. We demonstrated that c-Rel is a negative prognostic factor for survival in patients with PDAC. Tumors with elevated c-Rel levels are characterized by an undifferentiated morphology with Epithelial-mesenchymal plasticity (EMP), contractility, and ECM remodeling. Specifically, tumors with high c-Rel levels have increased fibronectin (FN1)–Integrin β3 (CD61) production, allowing them to have increased tolerance to isolation stress and metastatic homing. Overall, our findings suggest a novel oncogenic role for c-Rel in PDAC.

## c-Rel is expressed in both human and murine PDAC

To assess the ubiquity of c-Rel in PDAC, we analyzed various human and mouse samples. c-Rel was expressed in all cell lines isolated from human and mouse PDAC (Suppl. Figure 1A). Notably, mouse PDAC cell lines were isolated from GEMMs (CK: *Ptf1a-Cre Kras*^*G12D*^; CKP: *Trp53*^*f/f*^ *Ptf1a-Cre Kras*^*G12D*^). We generated a mouse PDAC TMA composed of 32 primary tumors with CK, CKP and CKPhet (*Trp53*^*f/+*^ *Ptf1a-Cre Kras*^*G12D*^) backgrounds and spontaneously formed metastatic tissues from liver (*n* = 4) and lung (*n* = 3). c-Rel was highly expressed in cancer cells and other TME components in all of the tumors (Suppl. Figure 1B).

**Fig. 1:**
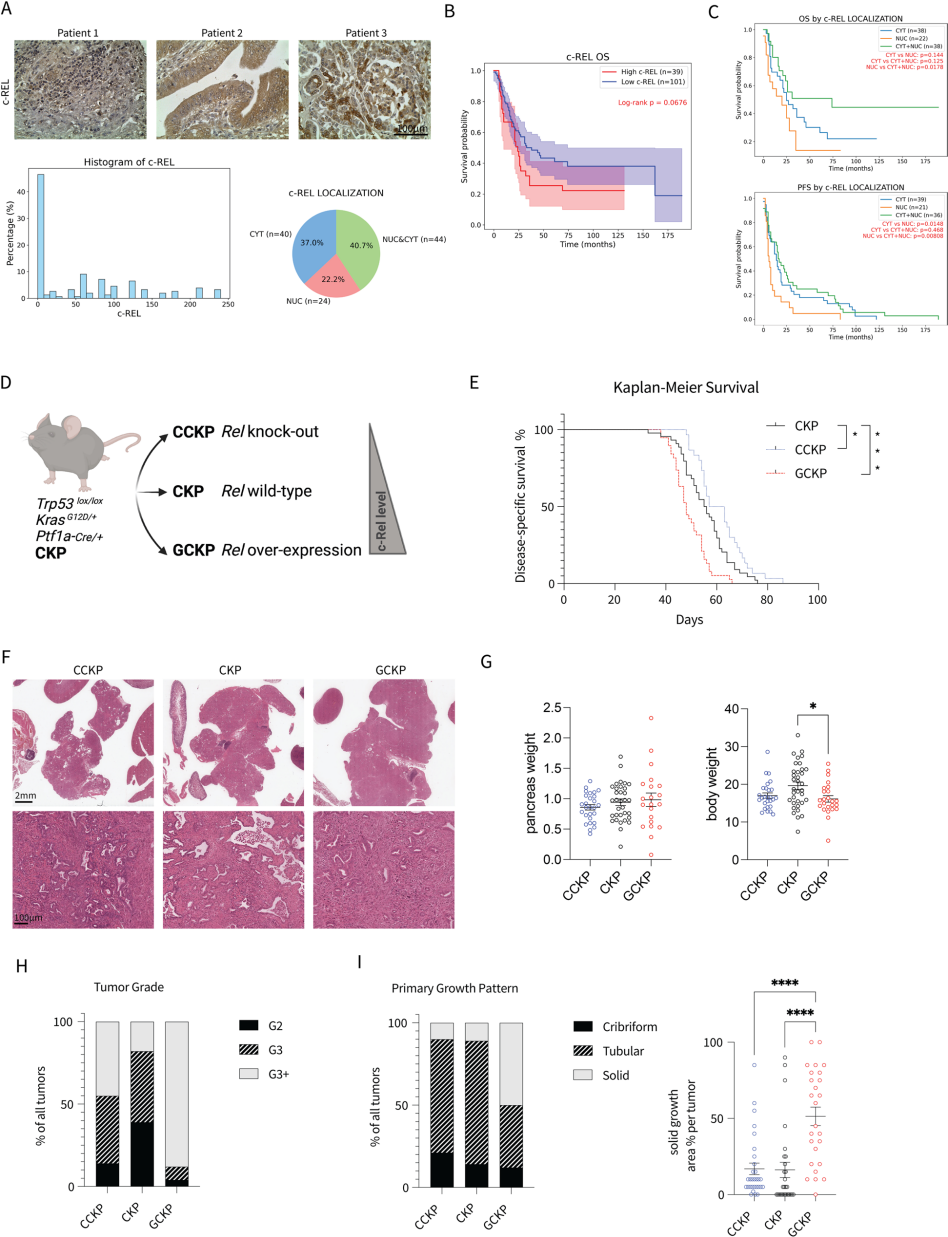
**A** Representative c-Rel IHC images of patient PDAC samples. The H-score distribution is shown in the histogram plot along with the pie chart displaying the percentage of c-Rel subcellular localizations. **B** Overall survival (OS) plot of patients based on c-Rel H-score. **C** OS and progression-free survival (PFS) plots for patients divided based on c-Rel subcellular localization. **D** Illustration of the generation of compound mutant mice with *Rel* knockout (CCKP), wild-type (WT) (CKP), and overexpression (GCKP). **E** Kaplan‒Meier survival curves for CCKP (n=30), CKP (n=44), and GCKP (n=38) mice. Statistical analysis was performed with the log-rank (Mantel‒Cox) test. **F** Representative histology sections for H&E staining. G) Pancreas (n.s.) and body weight (p=0.0172) comparisons. **H**, **I** Chi-square test was used for tumor grade (p<0.0001) and primary growth pattern (p<0.0001) analysis. For the solid growth area % per tumor, ordinary one-way ANOVA (p<0.0001) and Tukey's multiple comparisons test were used. The number of tumor-bearing mice analyzed by a pathologist was as follows: CCKP, n= 29; CKP, n=28; and GCKP, n=26. Unless otherwise indicated, for all analyses, ordinary one-way ANOVA was used. ANOVA p values are given in the figure legends. Tukey's multiple comparison tests are shown in the figures

In a cohort of resected human PDAC patients, c-Rel protein expression was noted in 108 out of 174 patients (Fig. 1A). 37% of the patients showed c-Rel localized only in the cytoplasm, with rest seen only in nucleus or in both nucleus and cytoplasm (Fig. 1A). A trend for a worse overall survival was observed for patients with high c-Rel expression (Fig. 1B). Moreover, especially patients with only nuclear c-Rel expression had the worst overall and progression-free survivals (Fig. 1C). Despite the survival effect, there was no c-Rel impact on clinical parameters (Suppl. Figure 1D). We further analyzed the TCGA, CPTAC-3, and Bailey RNAseq datasets via the pdacR online tool [34]. We categorized patients into low vs. high *REL* expression groups and used cut-off values of 0.5, 0.65, and 0.75 to compare the top-median, top-tertile, and top-quartile groups, respectively, with the other groups. Although most of the findings were statistically insignificant, a trend toward poorer prognosis in patients with high *REL* expression was observed in the TCGA and Bailey cohorts (Suppl. Figure 1C). These results suggest an oncogenic function of c-Rel in PDAC survival.

## Higher c-Rel levels drive partial EMT and activate the stroma in PDAC

To study c-Rel function more closely in PDAC via GEMMs, we generated compound mutant mice by crossing CKP mice with either a *Rel*^f/f^ knockout model (CCKP) or a *GFP-Rel*^*f/+*^ overexpression model (GCKP) (Fig. 1D). In CCKP, once the *Rel*^f/f^ allele is recombined, Exon-1 of *Rel* gene is removed and a promoterless EGFP cassette meets with a PGK promoter allowing constitutive production of the reporter signal [35]. In the GCKP model, the transgenic *Rel* is tagged with EGFP on the N’ termini. The Cre recombination removes the *STOP* cassette, allowing constitutive production of EGFP-tagged c-Rel under the CAG promoter [36]. Notably, all the mice were screened for possible recombination of the *floxed* alleles in the body, since *Ptf1a-Cre* can cause recombination in the paternal germline with high efficiency [37]. c-Rel IHC proves the absence and the overexpression of c-Rel in cancer cells in the CCKP and the GCKP models, respectively (Suppl. Figure 1E). Of note, we observed strong c-Rel signal in stromal cells as illustrated in the CCKP image.

c-Rel expression was inversely correlated with the survival: GCKP mice had a shorter lifespan, whereas CCKP mice survived longer (Fig. 1E). Although their tumor size wasn’t different, GCKP mice had lower body weight, indicating a worse prognosis (Fig. 1G). The GCKP tumors had more undifferentiated morphology (Fig. 1F). In particular, the GCKP tumors exhibited a higher tumor grade (Fig. 1H) and solid growth pattern (Fig. 1I), as blindly assessed by a pathologist.

**Fig. 2:**
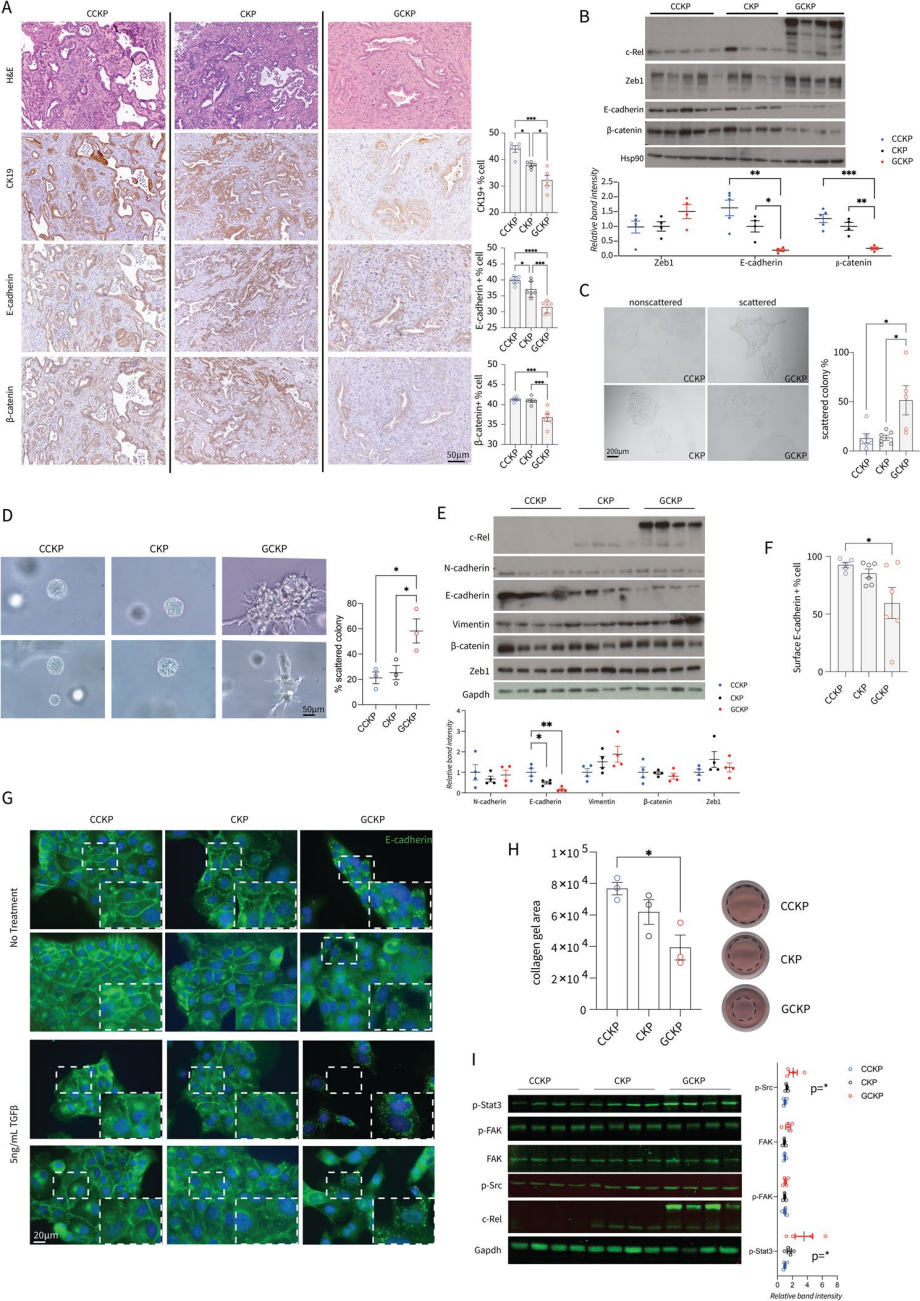
**A** Representative H&E and IHC images of the respective targets and their quantification. (CK19 p=0.0002, E-cad p<0.0001, and β-cat p=0.0002). **B** Western blot analyses of bulk tumor protein lysates. HSP90 was used as a loading control. The blots are quantified and normalized to HSP90 (Zeb1 n.s., E-cad p=0.002, β-cat p=0.0004) **C** Representative scattered and non-scattered colony morphologies and their quantification under a brightfield microscope (p=0.0085).** D** Representative images of organoids in Matrigel from CCKP, CKP, and GCKP cells. The scattered colonies are quantified and plotted as given (p=0.0172). **E**) Western blot analyses of isolated cancer cells. The signal is normalized to Gapdh (E-cad p=0.0021, Vim n.s. but test for linear trend p=0.0451, N-cad, β-cat, Zeb1 n.s.). **F** Surface E-cadherin expression was assessed by flow cytometry (p=0.0424). **G** Representative images of E-cadherin immunofluorescence staining are shown. The cells were either not treated or treated daily with 5 ng/mL TGF for two days **H** Collagen gels were visualized 4 days after seeding and quantified for surface area (p=0.0228). **I** Western blot analyses and their quantification with lysates from isolated cancer cells at 70% confluency under adherent conditions (Test for a linear trend p-Src p=0.0358, FAK n.s., p-FAK p= 0.708, p-Stat3 p=0.0307). Unless otherwise indicated, for all analyses, ordinary one-way ANOVA was used. ANOVA p values are given in the figure legends. Tukey's multiple comparison tests are shown in the figures

On the basis of tumor histopathology, we analyzed EMP. IHC (Fig. 2A), immunoblotting (Fig. 2B), and RT‒qPCR (Suppl. Figure 2A) of bulk tumor tissues revealed a trend towards decrease in the expression of epithelial markers (E-cadherin, CK-19, and β-catenin) and increase in the expression of mesenchymal markers (Vimentin, Zeb1, Zeb2, and Snai2), with a modest reduction in *Snai1* at the transcriptional level. Cancer cells isolated from primary tumors presented greater morphological scattering in vitro (Fig. 2 C) and in organoid cultures in Matrigel (Fig. 2D). Immunoblot analyses showed decreased E-cadherin and increased Vimentin expression with higher c-Rel (Fig. 2E). GCKP cells presented reduced surface E-cadherin expression (Fig. 2F) and displayed intracellular E-cadherin speckles aligning with partial-EMT characteristics (Fig. 2G) [38]. The difference in EMT observed between CCKP and CKP tumors in vivo was not evident in vitro under basal conditions, but TGFβ treatment potentiated this difference. Under TGFβ treatment, compared with CKP, CCKP cells resisted to surface E-cadherin expression reduction (Suppl. Figure 2B). Multiple previously published EMT-related signatures correlated positively with *REL* in PDAC samples, as analyzed in the EMTome database (Suppl. Figure 2C) [39]. Moreover, additional PDAC patient datasets were divided from the median based on *REL* expression. The *HALLMARK_EMT* signature was significantly enriched in REL-high samples (Suppl. Figure 2D). In parallel, c-Rel expression enhanced cancer cell contractility (Fig. 2H), with increased Src and Stat3 phosphorylation and no difference in p-FAK (Fig. 2I). Nevertheless, the GCKP cells were more sensitive to dasatinib (Src-i) than to defactinib (FAK-i) in terms of viability (Suppl. Figure 2E).

**Fig. 3:**
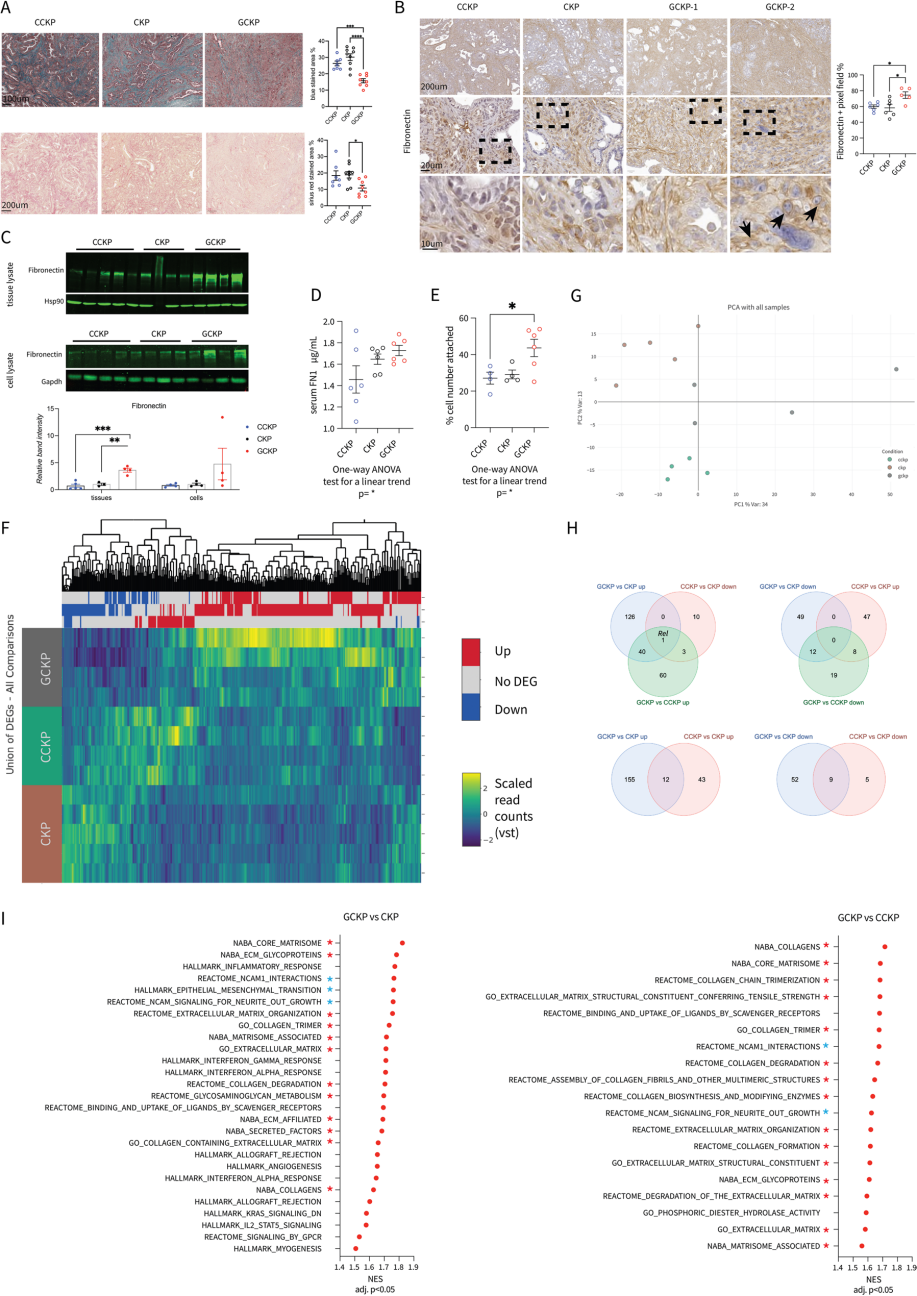
**A** Representative images of Masson's trichrome (p<0.0001) and Picosirius-red stained tumors and their quantification (p=0.0248) **B** Representative FN1-IHC images (p=0.0186). GCKP-1 reflects fibrillar FN1 deposition around cancer cells individually. GCKP-2 reflects the perinuclear FN1 signal intracellularly, as shown with arrowheads.**C** Western blot for FN1 in bulk tumors and isolated cancer cell lysates. The expression is normalized to HSP90 (tissues p=0.0002, cells n.s.) **D** Mouse serum FN1 ELISA results (One-way ANOVA for a linear trend p=0.0366) **E** Fibronectin adhesion assay. The number of cells attached to the FN1-coated wells was normalized to the seeding number (One-way ANOVA test for a linear trend p=0.0242) **F** Heatmap of the differentially expressed genes in the RNA-seq analysis of CCKP-CKP-GCKP cells. **G** Principal component analysis (PCA) plot of the cells used for the RNAseq analysis. **H** Venn diagrams illustrating the number of common genes up- or down-regulated in the given comparison pairs.** I** GSEA of the DEGs between the given comparison pairs. The red stars indicate enrichments related to ECM remodeling, whereas the blue stars indicate EMT-related pathways. Unless otherwise indicated, for all analyses, ordinary one-way ANOVA was used. ANOVA p values are given in the figure legends. Tukey's multiple comparison tests are shown in the figures

Further histopathological examination revealed notable changes in the ECM. GCKP tumors presented less collagen deposition (Fig. 3A). Conversely, FN1 expression, which was particularly elevated in GCKP tumors, was confirmed through IHC (Fig. 3B) and immunoblotting (Fig. 3C). In CCKP and CKP tumors, FN1 primarily surrounded collective tubular-cribriform structures, whereas in GCKP tumors, it individually encircled undifferentiated cancer cells, as evidenced in representative images of GCKP-1 cells (Fig. 3B). Intriguingly, some cancer cells displayed a perinuclear FN1 pattern, as exemplified in GCKP-2 (Fig. 3B) cells. The serum FN1 level increased with increasing c-Rel levels in primary PDAC tumors (Fig. 3D). Additionally, c-Rel enhanced FN1 responsiveness, as GCKP cells attached to FN1 faster (Fig. 3E).

To corroborate our findings, we performed RNA-seq using isolated CCKP, CKP, and GCKP cancer cells, with pairwise comparisons for the GCKP vs. CKP, GCKP vs. CCKP, and CCKP vs. CKP samples (Fig. 3F). Heterogeneity in cancer is the norm, as exemplified by the PCA of the cancer cell lines (Fig. 3G). Nevertheless, cells with the same genotypic background were grouped (Fig. 3G). To our surprise, except *Rel* there were no common genes that were differentially and oppositely expressed between the GCKP vs. CKP and, CCKP vs. CKP cells (Fig. 3H). On the other hand, 12 and 9 common genes were up- and downregulated, respectively, in the GCKP and CCKP cells compared with the CKP cells (Fig. 3H).

We attributed the absence of common genes in the c-Rel overexpression and knockout settings to the differential regulation of NF-κB proteins. Therefore, we checked the NF-κB dynamics in various settings. Under basal conditions, c-Rel was present mostly in the cytoplasm, whereas the canonical NF-κB inducer TNFα quickly facilitated its nuclear translocation (Suppl. Figure 3A). Notably, only a 20-minute treatment with the nuclear export inhibitor leptomycin-B was sufficient to accumulate c-Rel in the nucleus (Suppl. Figure 3A). Coimmunoprecipitation analysis showed c-Rel and RelA heterodimerization (Suppl. Figure 3B). Moreover, compared to controls, although the total c-Rel and RelB expression was reduced in RelA knock-out cells (Suppl. Figure 3C), total RelA and RelB expression didn’t change in CCKP or GCKP cells (Suppl. Figure 3D). We observed a notable and gradual increase in IKBα, IKBβ, p105 and p52 proteins from CCKP to GCKP cells. Despite the increase in p105 expression, p50 levels remained in the GCKP (Suppl. Figure 3D). Opposite to the total expression, c-Rel nuclear translocation was increased in RelA-knockout CK (CKA) and CKP cells (CKPA) compared to controls (Suppl. Figure 3E). Under the basal conditions, the nuclear translocation of RelA, RelB, p50 and p52 were decreased in both CCKP and GCKP cells compared to CKP. These results suggest that the crosstalk between c-Rel and RelA is complex and context dependent.

In line with our results, multiple gene sets related to EMT and ECM remodeling were enriched in GCKP cells (Fig. 3I). Additionally, *REL* expression was correlated with Moffitt´s activated stroma signatures in multiple human PDAC transcriptomics datasets (Suppl. Figure 3G). Previously, a detailed molecular analysis revealed that PDAC tumors with multiple stromal subtypes (subTMEs) coexist [40]. Tumors with reactive subTMEs had higher c-Rel and FN1 protein expression in tumor cells and stroma, respectively (Suppl. Figure 3H). In parallel with these findings, c-Rel protein in tumor cells was positively correlated with the presence of FN1 in the stroma (Suppl. Figure 3I). Notably, integrin signaling associated with FN1 binding was also enriched in PID (Pathway interaction database) in GCKP cells (Suppl. Figure 3J). GSEA of human samples revealed significant enrichment of ECM-receptor interactions, particularly integrins, in REL-high samples (Suppl. Figure 3K). Finally, we analyzed 2 human PDAC scRNA-seq datasets, each pooled with multiple patient samples. As expected, *REL* expression was mostly observed in immune cells, such as myeloid cells, T/NK cells, B/plasma cells and mast cells (Suppl. Figure 3L). The cell types were distinguished on the basis of the expression of target genes given in the tabular results (Suppl. Figure 3L). The analysis revealed that epithelial cells with a high *REL* had a greater fraction of cells expressing multiple integrin family transcripts with greater expression (Suppl. Figure 3L). Overall, c-Rel regulates EMT and ECM remodeling, possibly involving FN1–integrin signaling in PDAC.

## c-Rel enhances isolation stress adaptation in PDAC cells

**Fig. 4:**
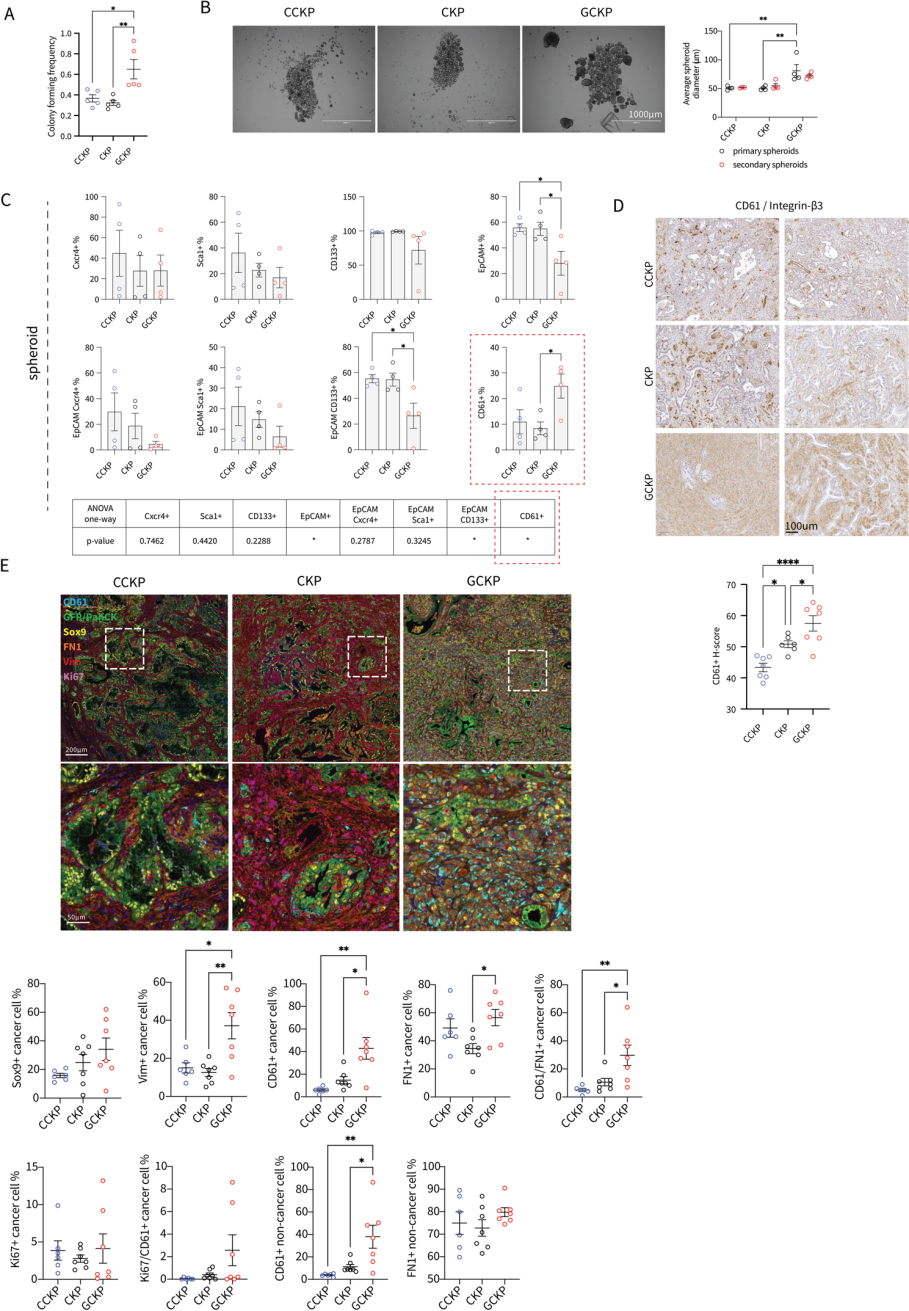
**A** Colony-forming frequency was assessed by the ability of cells to form an organoid in Matrigel (one-way ANOVA, p=0.0041). Tukey's multiple comparisons test results are shown in the figure. **B** Representative microscopy images of spheroids formed under nonadherent condition. The average spheroid diameter was quantified and analyzed by two-way ANOVA (genotype factor p=0.007). Šídák's multiple comparison tests between genotypes are shown in the figure.** C** Surface expression of selected proteins on spheroids as assessed by flow cytometry analysis. p values for the one-way ANOVA test are given in the table below. **D** Representative IHC images of CD61/integrin-3 and their quantification (one-way ANOVA, p=0.0001). **E** A representative image for multiplex immunofluorescence (IF) staining of CCKP, CKP and GCKP tissues and the quantification plots (one-way ANOVA, in cancer cells: Sox9+ test for a trend p=0.0478, Vim+ p=0.0022, CD61+ p=0.0015, Ki67+ p=n.s., FN1+ p=0.0241, FN1/CD61+ p=0.0047, Ki67/CD61+ p=0.0478, in non-cancer cells: CD61+ p=0.0035, FN1+ p=n.s.)

Previously, we reported a reduction in the *NFKB_SIGNALING* signature in human PDAC spheroids under nonadherent conditions compared with their adherent counterparts [41]. In contrast, the *REL* mRNA level was increased, unlike that of the signature (Suppl. Figure 4 A). In parallel, among the NF-κB proteins, only c-Rel and RelA consistently increased protein expression in spheroid culture (Suppl. Figure 4B). On the basis of these findings, we conducted multiple assays to measure c-Rel-induced resistance to isolation stress. GCKP cells demonstrated greater colony-forming frequency, as quantified by their ability to form organoids in Matrigel (Fig. 4A), and formed larger spheroids under adherence-free conditions (Fig. 4B). We tested the surface expression of predefined stemness-associated markers, including EpCAM, Cxcr4, CD44, CD133, Sca1, and CD61 (integrin β3), in adherent (Suppl. Figure 4C) and nonadherent serum-free spheroid cultures (Fig. 4C). To our surprise, all the markers, either individually or in combination with EpCAM, were reduced in GCKP cells, except for CD61 in spheroid cultures (Fig. 4C). In parallel, CD61 expression was increased with c-Rel levels in primary tumors (Fig. 4D). Overall, these results suggest that c-Rel is involved in CD61-dependent cell survival under isolation stress.

## Ectopically expressed c-Rel increases EMP, ECM remodeling, and resistance to isolation stress in cancer cells

To validate our findings regarding the regulatory role of c-Rel in multiple aspects of PDAC behavior, we utilized the auxin-inducible degron 2 (AID2) system (Suppl. Figure 4D) [42]. The AID2 system allows users to rapidly knock down their protein of interest after auxin analog (5phIAA) treatment. In two CCKP cell lines (A2929 and A2770), we overexpressed c-Rel with both GFP and mAID tags (Suppl. Figure 4E), enabling rapid degradation of c-Rel within 2 h of 5phIAA treatment (Suppl. Figure 4 F). Compared with mock-transfected cells, Rel-pAY15-transfected cells presented a scattered cellular morphology with reduced surface E-cadherin expression with or without TGFβ induction (Suppl. Figure 4G). The increased spheroid size in Rel-pAY15 cells was reversed with 5phIAA-induced c-Rel degradation (Suppl. Figure 4H). Moreover, the surface expression of CD61 followed a similar trend, rescuing the phenotype (Suppl. Figure 4I). Notably, although not significantly, EpCAM and CD133 surface expression increased after c-Rel degradation (Suppl. Figure 4J).

Further exploring our previous findings, we performed a multiplex immunofluorescence (IF) staining on primary CCKP, CKP and GCKP tumors (Fig. 4E). The panel was composed of CD61, GFP/PanCK, Sox9, FN1, Vimentin and Ki67 (Suppl. Figure 4K). To specifically capture the undifferentiated cancer cells, along with the GFP/PanCK, Sox9 staining was used. As expected, we observed higher numbers of Sox9+, Vim+, CD61+, FN1 + and CD61/FN1 double + cancer cells in GCKP tumors. Although the proliferation wasn’t different in all cancer cells, GCKP tumors had more proliferating CD61 + cancer cells (CD61/Ki67 double+). The expression of CD61 was also higher in cells other than the cancer cells. Despite the trend, FN1 expression change in non-cancer cells wasn’t significant.

**Fig. 5:**
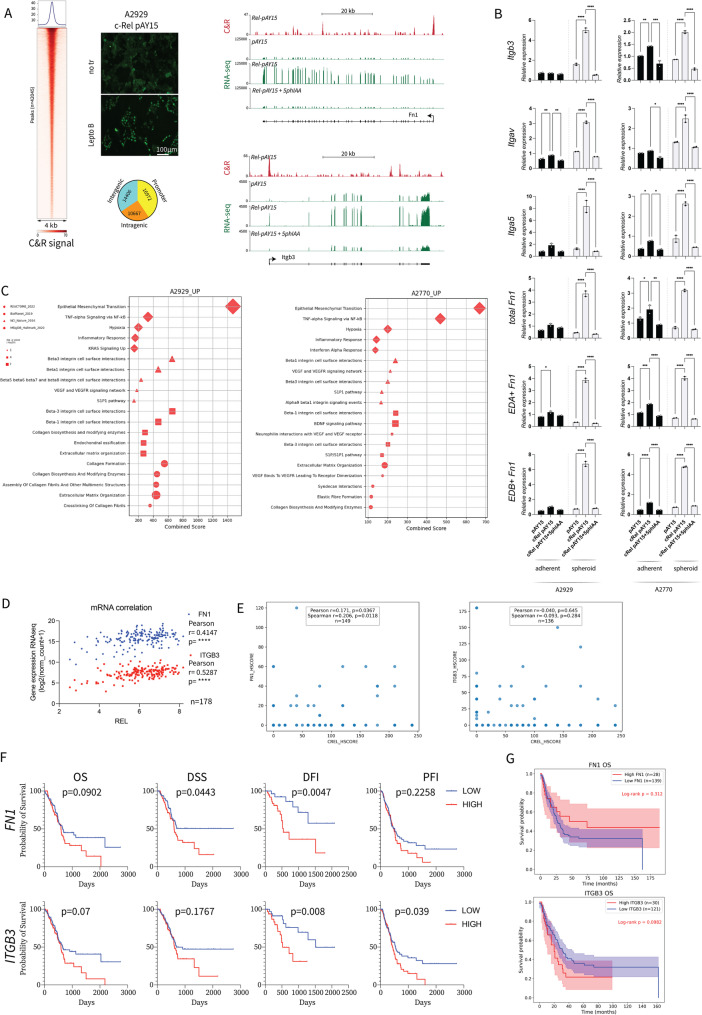
**A** CUT&RUN analysis to assess c-Rel genomic binding. Fluorescence microscopy imaging of the Rel-pAY15 stably transfected A2929 cell line. c-Rel is tagged with GFP. Lepto-B: Leptomycin-B. Heatmap showing the genomic regions differentially bound by c-Rel. The peak numbers for the genomic locations where c-Rel is bound are given in a pie chart. The CUT&RUN and RNAseq tracks display the expression levels of the *Fn1 *and *Itgb3* genes in mock-transfected (pAY15), c-Rel-overexpressing (Rel-pAY15), and degron-induced c-Rel-overexpressing A2929 (Rel-pAY15 + 5phIAA) cell lines. **B** Normalized RT‒qPCR analysis for the selected targets. Stably transfected cells are cultured under adherent (2D) or nonadherent conditions (3D) (A2929 c-Rel expression factor, *Itgb3* p= 0.0006*, ItgaV* p=0.0001*, Itga5* p=0.0069*, total Fn1* p=0.0014*, EDA+ Fn1* p<0.0001*, EDB+ Fn1* p=0.0004; for A2770 c-Rel expression factor* Itgb3* p=0.0011*, ItgaV* p=0.0032*, Itga5* p=0.0014*, total Fn1* p=0.0027*, EDA+ Fn1* p<0.0001*, EDB+ Fn1* p<0.0001). **C** Overrepresentation analysis performed by *Enrichr* for Rel-pAY15 cells. The analysis is performed only with the genes that were differentially expressed in Rel-pAY15 cells compared with those in pAY15-only cells or in Rel-pAY15-induced degron cells. From each set, only the top 5 hits are selected on the basis of their combined scores. **D** Correlation of the *REL *with *FN1* and *ITGB3* mRNA expression in primary tumor samples from the TCGA_PAAD cohort. The graphs are adapted with data from the UCSC-Xena TCGA PAAD dataset. The Pearson r correlations are displayed in the figure for each target. **E** Patient TMA H-score correlation plots for c-Rel versus ITGB3 or FN1. F) Kaplan–Meier survival curves for patients in the TCGA dataset, divided by the median expression level, on the basis of either *FN1* or *ITGB3* expression. OS: overall survival, n= 178; DSS: disease-specific survival, n=172; DFI: disease-free interval, n=69; PFI: progression-free interval, n=178. The graphs are adapted with data from the UCSC-Xena TCGA PAAD dataset. **G** Patient TMA overall survival curves based on ITGB3 and FN1 H-scores. Statistical analysis is performed with the log-rank (Mantel‒Cox) test. Unless otherwise indicated, for all analyses, two-way ANOVA is used. Šídák's multiple comparison tests between genotypes are shown in the figures

To assess the direct transcriptional regulation of c-Rel in the associated phenotypes, we performed a CUT&RUN experiment with the degron cells [43, 44]. CUT&RUN is an alternative to ChIP, where antibody binding to target proteins allows micrococcal nuclease recruitment and DNA cleavage, releasing target‒protein&DNA complexes. The accumulation of c-Rel in the nucleus was achieved with the nuclear export inhibitor leptomycin-B (Fig. 5A). The peaks indicated broad c-Rel binding to intergenic, intragenic, and promoter regions (Fig. 5A). Analysis of the *FN1* and *Itgb3* genes suggested that c-Rel binds to their promoter regions (Fig. 5A). The mRNA levels of *FN1* and several RGD integrins (*Itgb3*,* ItgaV*,* Itga5*), as assessed via RT‒qPCR, were elevated in c-Rel-overexpressing cells, especially under 3D spheroid culture conditions, and returned to lower levels following 5phIAA-induced c-Rel degradation (Fig. 5B). Therefore, we performed RNA-seq analysis on degron cells in spheroid cultures. In parallel with previous findings, cells overexpressing c-Rel presented significant upregulation of *FN1* and *Itgb3* mRNA levels (Fig. 5A). The increase was reversed after 5phIAA treatment (Fig. 5A). Moreover, the overrepresentation analysis performed with Enrichr [45–47] showed enrichment for the EMT, Itgb3 signaling, and ECM modeling pathways, which were rescued by the degron stimulation (Fig. 5C). *REL* mRNA levels were positively correlated with both *FN1* and *ITGB3* levels in the TCGA dataset (Fig. 5D). Both *FN1* and *ITGB3* had prognostic value in multiple survival analyses (Fig. 5F).

The EMT regulators *Snai1*,* Snai2*,* Tgfb1 and Zeb2*, and other integrins *Itga5* and *Itgav* were also among the c-Rel DNA-binding targets (Suppl. Figure 5A). The expression of these genes, with the exception of TGFB1, was positively correlated with *REL* in the TCGA cohort (Suppl. Figure 5B). *ITGAV* but not *ITGA5* showed negative prognostic impact in patient survival in the TCGA cohort (Suppl. Figure 5C).

In patient TMA, c-Rel expression correlated with FN1 (Fig. 5E) and ITGA5 (Suppl. Figure 5E). Both ITGB3 (Fig. 5G) and ITGA5 (Suppl. Figure 5E) showed a negative prognostic impact in trend for patient OS. Moreover, c-Rel correlated with the Itgb3 expression in mouse CKP tumors (Suppl. Figure 5D). We further analyzed whether any clinical parameter shows a difference related to ITGB3, ITGA5 and FN1 expression. Patients with more extracapsular extension had more ITGB3 expression in their primary tumors (Suppl. Figure 5F). Although not significant, patients with metastasis in continuity showed more ITGA5 in primary tumors (Suppl. Figure 5F).

Notably, some genes where c-Rel occupied the promoters were downregulated according to the results of the transcriptomic analyses (Suppl. Figure 5G). Further overrepresentation analysis revealed significant downregulation for various metabolic pathways when c-Rel was overexpressed (Suppl. Figure 5H). Our findings demonstrate the direct involvement of c-Rel in EMT and FN1-integrin signaling.

## Fibronectin depletion in cancer cells does not affect EMT or survival

**Fig. 6:**
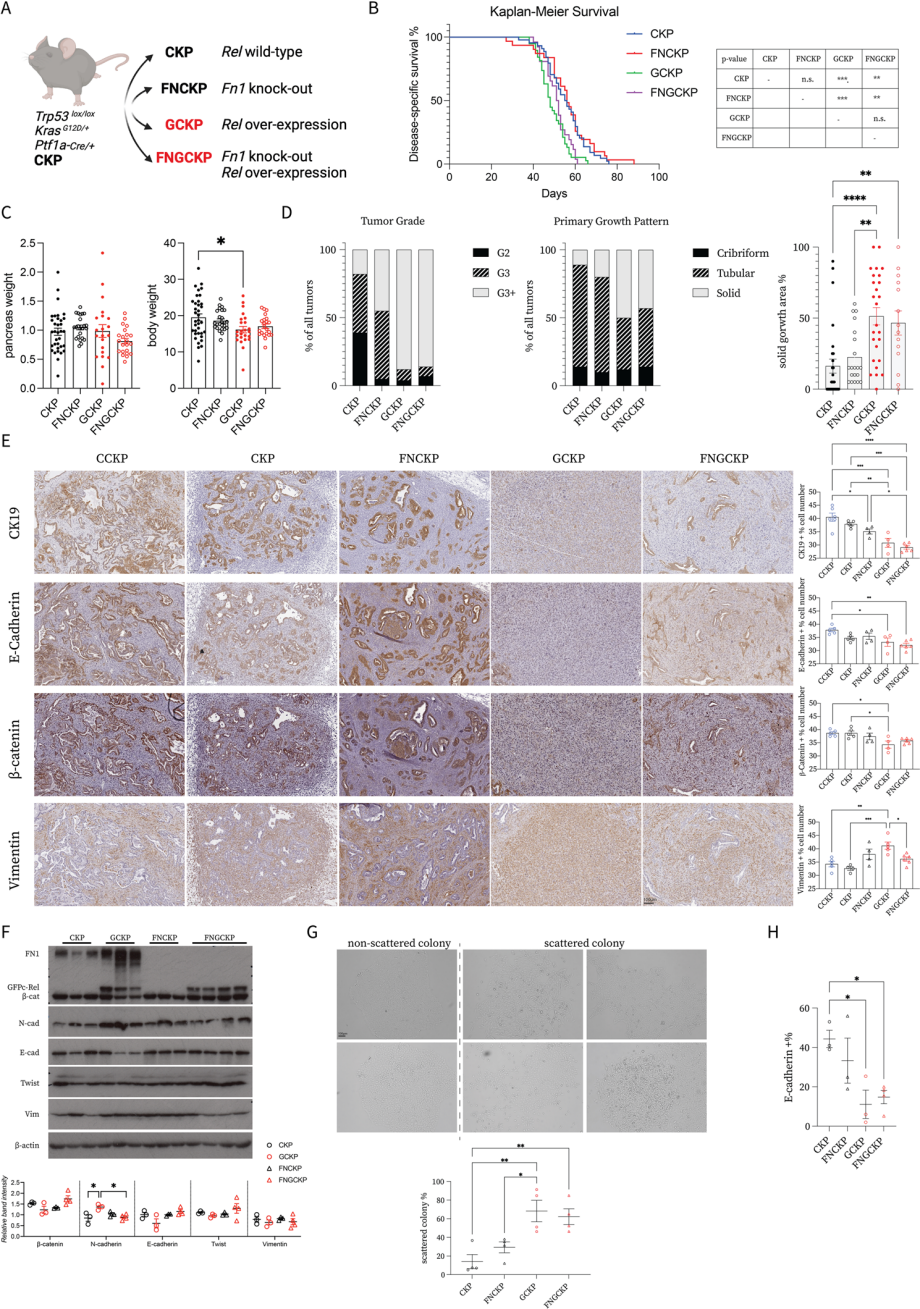
**A** Schematic illustrating the generation of compound mutant mice. **B** Kaplan‒Meier survival curves for the mice (CKP, n=44; FNCKP, n=31; GCKP, n=38; and FNGCKP, n=26). Statistical analysis was performed with the log-rank (Mantel‒Cox) test in pairs, and p values are displayed in the table. **C** Pancreas (n.s.) and body weight (p=0.0214) comparisons. **D** Chi-square test (p<0.0001) was used for tumor grade and primary growth pattern analysis. For the solid growth area % per tumor, ordinary one-way ANOVA (p<0.0001) and Tukey's multiple comparisons test were used. The number of tumor-bearing mice analyzed was as follows: CKP, n=28; FNCKP, n=20; GCKP, n=26; and FNGCKP, n=14. **E** Representative IHC images of the given EMT markers and their quantification (CK19 p<0.0001, E-cadherin p=0.0028 and -catenin p=0.0087, vimentin p=0.0008). The scale bar is 100mm. **F** Immunoblot analyses of several EMT markers in isolated cancer cells. Signal is normalized to -Actin (-cat p=0.055, N-cad p=0.0186, E-cad p=0.0496, Twist and Vim n.s.) **G** Representative scattered and non-scattered colony morphologies and their quantification under a brightfield microscope (p=0.002). H) Surface E-cadherin expression was assessed by flow cytometry (p=0.0238). Unless otherwise indicated, for all analyses, ordinary one-way ANOVA was used. ANOVA p values are given in the figure legends. Tukey's multiple comparison tests are shown in the figures.

Given the positive regulatory effect of c-Rel on the FN1–integrin axis both in vivo and in vitro, we hypothesized that the absence of FN1 in cancer cells might counteract the c-Rel-induced phenotypes. We generated compound mutant mice by crossing the* Fn1*^*f/f*^ model with CKP and GCKP mice (Fig. 6A). The reduced overall survival (OS) in GCKP mice was not restored to longer CKP durations (Fig. 6B). There was also no difference in survival between CKP and FNCKP mice (Fig. 6B). Neither pancreatic, nor the body weight showed a difference after FN1 deletion (Fig. 6C). Notably, we validated the reduction in FN1 expression in tumor tissues via RT‒qPCR (Suppl. Figure 6A), immunoblotting (Suppl. Figure 6B), and IHC (Suppl. Figure 6C). FN1 can be found in soluble and insoluble forms. Soluble FN1 is mostly produced by the liver and found in the blood. The insoluble form is named as cellular fibronectin and produced by various cell types. The cellular FN1 contains EDA and/or EDB domains, while plasma FN1 lacks them. The number of cellular FN1 isoforms with EDA and EDB domains also decreased under the FN1 knockout conditions (Suppl. Figure 6 A). Although FN1 was depleted in cancer cells, it remained abundant in the ECM (Suppl. Figure 6C). Moreover, FNGCKP tumors still maintained high-grade tumors with major solid growth patterns similar to those of GCKP tumors (Fig. 6D). Compared with CKP tumors, FNCKP tumors had a higher tumor grade, although their growth pattern morphology was not different (Fig. 6D). Moreover, FN1 depletion in cancer cells rescued the EMT in vivo in neither CKP nor GCKP tumors. Compared with those in the control CKP and GCKP samples, epithelial CK19, E-cadherin, and β-catenin and mesenchymal vimentin protein expression did not differ between the FNCKP and FNGCKP samples (Fig. 6E). To eliminate FN1 assistance from stromal sources during EMT in vivo, we analyzed cancer cells in vitro. The isolated cancer cells were validated for their lack of FN1 and overexpression of c-Rel (Fig. 6F). The absence of FN1 rescued the scattered morphology observed in neither CKP nor GCKP cells (Fig. 6G). Compared with CKP cells, FNCKP cells presented no consistent changes in EMT protein marker expression (Fig. 6H). In FNGCKP cells compared to GCKP, the E-cadherin was trended to increase, while the N-cadherin was decreased (Fig. 6F). However, the surface expression of E-cadherin did not change in the CKP-FNCKP and GCKP-FNGCKP comparisons (Fig. 6H). Therefore, FN1 is not essential for EMT, and its absence cannot rescue the survival and EMT phenotypes observed in GCKP tumors.

## Fibronectin is necessary for c-Rel-induced isolation stress resistance in cancer cells

**Fig. 7:**
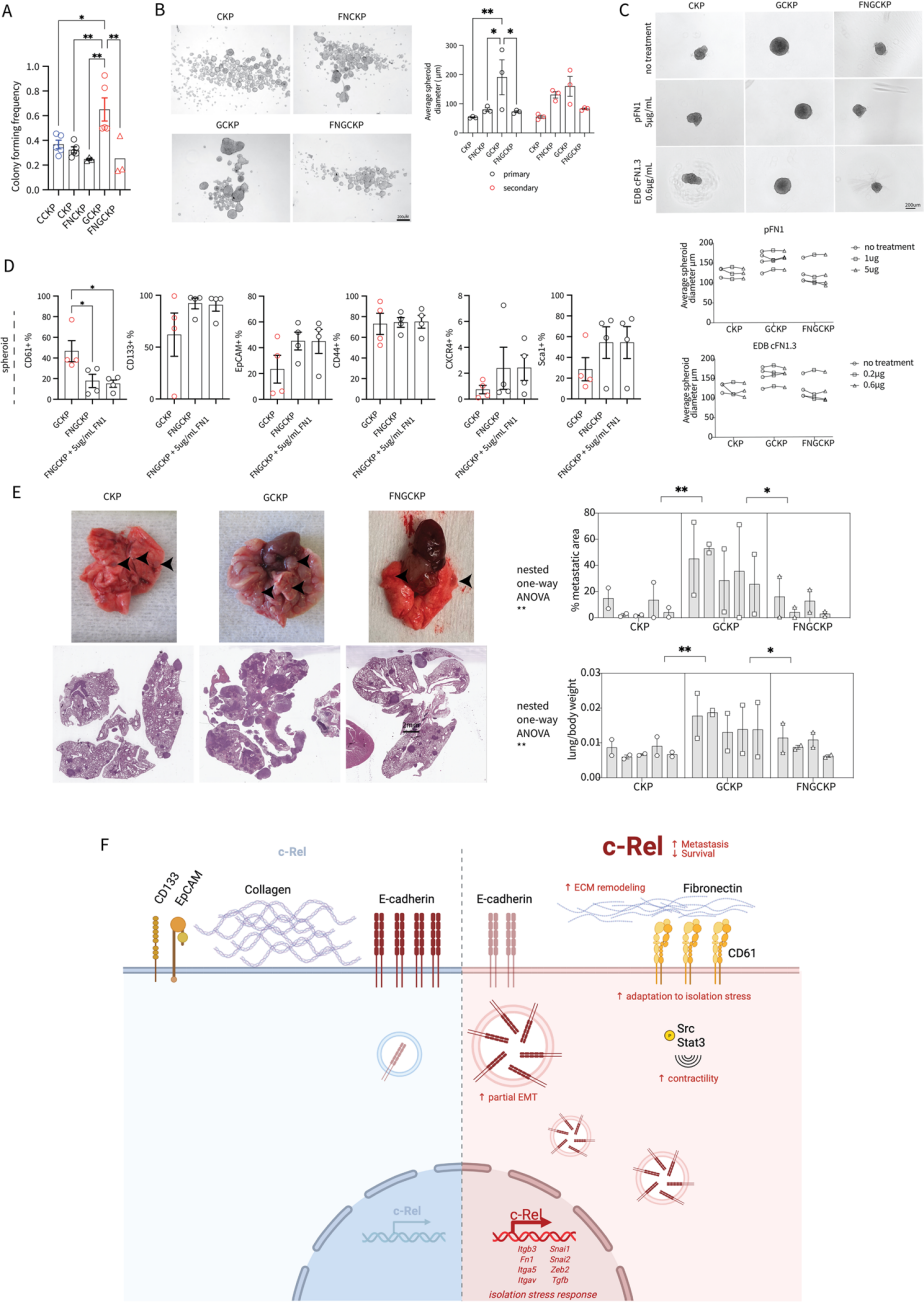
**A** Colony-forming frequency was assessed by the ability of cells to form an organoid in Matrigel (one-way ANOVA, p=0.0015). Tukey's multiple comparisons test results are shown in the figure. **B** Representative microscopy images of spheroids formed under nonadherent conditions. The average spheroid diameter was quantified and analyzed (genotype factor p=0.0227). **C** Representative spheroid images and their diameter quantification. The spheroid medium was supplemented with either human plasma fibronectin (pFN1) or cellular fibronectin with EDB domain + type II domains 8−14 (EDB cFN1.3) (treatment factor p= n.s.) **D** Surface expression of selected proteins on spheroids as assessed by flow cytometry analysis (one-way ANOVA test for CD61 p=0.0244, CD133, EpCAM, CD44, CXCR4, Sca1 p= n.s.). Tukey's multiple comparisons test results are shown in the figure. **E** Representative macroscopic and microscopic images of the transplanted mouse lungs. The number of cell lines used per genotype are CKP=5, GCKP=5 and FNGCKP=4. Each cell line was injected two times to two individual mice. Nested one-way ANOVA was used for both analyses. Tukey's multiple comparisons test results are shown in the figure. Unless otherwise indicated, for all analyses, two-way ANOVA was used. ANOVA p values are given in the figure or their legends. Šídák's multiple comparison tests between genotypes are shown in the figures.** F** Schematic depicting the function of c-Rel in PDAC pathophysiology. c-Rel is an oncogenic factor involved in PDAC survival and metastasis. Increased c-Rel expression remodels the ECM by converting the collagen-rich stroma to a fibronectin-rich stroma. Cancer cells undergo epithelial-to-mesenchymal transition, increasing EMP by reducing the surface expression of E-cadherin and contractility via Src and Stat3 phosphorylation. Once isolation stress is induced under nonadherent conditions, c-Rel induces a niche enriched with fibronectin, coupled with increased *Itgb3, Itga5* and *Itgav* expression. These changes are also supported by EMP, where c-Rel directly induces EMT-TFs such as *Snai1, Snai2, Zeb2, *and* Tgfb*. Increased tolerance to isolation stress ultimately drives metastasis. Created in BioRender. Kabacaoglu, D. (2025)

We wanted to test whether FN1 is required to maintain CD61-associated anchorage-independent growth in GCKP cells. Compared with that of the CKP control (Fig. 7A), the colony formation frequency of the FNCKP cells in Matrigel did not differ. On the other hand, a greater colony-forming frequency was rescued in GCKP cells after FN1 deletion (Fig. 7A). The greater average spheroid size of GCKP under adherent-free conditions was also rescued in the FNGCKP (Fig. 7B).

Intriguingly, neither plasma FN1 nor cellular FN1-EDB at various concentrations in culture medium rescued the spheroid size of the FNGCKP cells (Fig. 7C). In line with previous findings, in the FNGCKP spheroids, the surface expression of CD61 was reduced relative to that in the GCKP spheroids, and supplementing plasma FN1 again did not rescue the phenotype (Fig. 7D). Moreover, in primary tumors, the absence of FN1 reduced CD61 levels (Suppl. Figure 6D). Although not significant, the expression of the other conventional CSC markers was increased (Fig. 7D). Our results indicate the involvement of c-Rel in cellular fitness during isolation stress under adherence-free conditions, an important concept in the metastatic cascade. To assess the importance of the c-Rel-mediated integrin-FN1 signaling axis in the metastatic cascade, we utilized IV injection of cancer cells followed by lung histopathology analysis. As expected, both the relative metastatic colony area in the lungs and the relative lung weights were increased in the GCKP cells (Fig. 7E). Moreover, the absence of FN1 in GCKP cells rescued the metastatic phenotype to CKP levels. Neither FN1 nor CD61 levels were reduced in FNGCKP lung colonies compared to GCKP, although trended to decrease (Suppl. Figure 6E). The cancer cells remained FN1 negative in FNGCKP. However, there was still strong FN1 ECM deposition, likely supported by stromal cells (Suppl. Figure 6E). Overall, the c-Rel-FN1-integrin signaling axis is a novel regulator of the metastatic cascade.

## Discussion

Herein, we propose a mechanism in which c-Rel is involved in multiple oncogenic facets, collectively regulating the survival and metastatic behavior of PDAC (Fig. 7). By directly regulating the expression of EMT-related transcription factors, c-Rel can influence EMP in PDAC. With the transcriptional regulation of key ECM structural and modifier proteins, c-Rel finetunes ECM remodeling. Finally, by directly regulating FN1 and CD61/integrin β3 expression, c-Rel modulates the anchorage-independent growth and metastatic homing of PDAC.

The survival analyses obtained from patient samples reveal a negative prognostic impact of c-Rel in patient survival. However, we don’t see a profound impact in clinical parameters. The kinetic and dynamic heterogeneity in c-Rel nuclear translocation and transcriptional activation can reduce the confidence in patient sample analyses [14]. Indeed, only short-term inhibition of nuclear export via leptomycin-B was sufficient to accumulate c-Rel, especially in the nucleus, indicating very dynamic nuclear translocation (Suppl. Figure 3A). Although we see differences in survival, a snapshot of neither c-Rel levels nor its nuclear localization obtained from a single timepoint point can be suitable for fully assessing the potential contribution of c-Rel to patient prognosis.

The phenotypic differences observed in GCKP tumors also did not always negatively overlap with those observed in CCKP samples. Although the nuclear localization of c-Rel was greater in RelA knockout cells than in control cells, RelA nuclear localization was lower in both CCKP and GCKP cells. The reduced translocation in the CCKP can be due to the absence of c-Rel:RelA dimers. On the other hand, both IKBα and IKBβ increase in GCKP cells can hold RelA in the cytoplasm, reducing its nuclear localization. This does not necessarily mean that RelA is less functional as a transcription factor in CCKP or GCKP cells. Variables such as ligand type, temporal control of stimulation, nuclear translocation oscillation dynamics, downstream gene targets and transcriptional coregulatory proteins must be further elaborated to understand the crosstalk between c-Rel and RelA. Nevertheless, evidence suggests that both redundant and nonredundant functions of c-Rel, in some cases, fine-tune RelA transcriptional activation in a ligand-specific manner during inflammation [48–53]. Such an interplay may also be evident in epithelial cell behavior in PDAC, hindering the phenotypic difference observed in the c-Rel knockout setting. The lack of consistency in total expression and nuclear localization of most proteins highlights additional cross-talk mechanisms between them.

c-Rel regulates total and surface E-cadherin expression i*n vitro* under adherent conditions. However, other EMT markers were not profoundly changed upon c-Rel modulation. The impact of c-Rel on EMT markers was rather evident in vivo and in the spheroids of the isolated cancer cells. These results indicate that c-Rel impact on EMT can be potentiated with additional factors.

Cellular plasticity and niche interactions are among the emerging hallmarks of cancer cell stemness [54]. Heterogeneity in terms of cellular plasticity and niche interactions may impact CSC subtypes. For example, Lgr5+ colorectal CSCs have the ability to self-renew and differentiate into Lgr5- invasive and metastatic cancer cells [55]. Interestingly, no Lgr5+ CSCs were detected as CTCs in the circulation. Moreover, the Lgr5- CTCs presented no change in the expression of conventional CSC markers, such as CD133 or CD44. A lack of a CSC niche may force them to survive through alternative mechanisms. Once colonized into the liver, owing to their plasticity, Lgr5- cells can partially differentiate back into Lgr5+ CSCs. Similar findings have also been reported in skin cancer. Compared with EpCAM+ tumor cells, EpCAM- tumor cells have greater tumor-initiating and metastatic capacity [56]. Interestingly, EpCAM+ cells were enriched for epithelial signatures and CSC markers such as CD133, Sox2 and Aldh1. The EpCAM- counterpart was enriched for EMT and the ECM, including FN1. EpCAM- and mesenchymal-like cells in skin and mammary cancer were shown to possess a grade of EMT with the combination of CD106, CD51, and CD61 surface expression [57]. Most of these cells in the intermediate state have comparable TIC capacities, but their invasiveness and metastasis differ. Although we primarily tested the surface expression of CD61, the transcriptomics data indicated a c-Rel-dependent increase in all of these markers. In parallel, conventional CSC surface expression markers mostly showed a reduced expression pattern or no change in GCKP. Therefore, we suggest a model in which c-Rel induces a tumor quasi-mesenchymal CSC subtype [58] with EMP in PDAC, increasing both the tumor initiating capacity and the metastatic capacity.

Previously, CD61 was shown to be an upstream inducer of c-Rel activation in erlotinib-resistant CSCs [29]. In addition, FN1-CD61 expression under the control of c-Rel activation highlights a positive feedback loop. Moreover, CD61+ CTCs were identified as a subcluster of PDAC CTCs [10]. Additionally, during isolation stress, CD61/CD51 and CD29/CD49e were associated with resistance to cell death [8, 59]. Among these targets, not only CD61 and CD51 but also CD49e showed increased expression during anchorage-independent growth in c-Rel-overexpressing cells. Therefore, we suggest a central role for c-Rel transcriptional activation in the resilience of CTCs before metastatic colonization under anchorage-independent growth.

The absence of FN1 in GCKP tumors cannot rescue survival, which might be due to the abundance of FN1 in the ECM derived from stromal cells in primary tumors. However, cells isolated from these tumors display impaired metastatic colonization. These results suggest tumor autonomous redundancy for FN1 production in primary tumors. However, in CTCs with metastatic potential, FN1-coupled CD61 surface expression is important. Moreover, the intracellular production of FN1 stimulates anchorage-independent growth under isolation stress. A reduction in CD61 surface expression is also coupled with the absence of FN1. The absence of CD61 expression on the cell surface could be due to two reasons. Differences in posttranslational modifications (e.g., citrullination [60]) in supplemented soluble FN1 in spheroid medium might result in an inability to rescue the reduced spheroid formation in FN1 knockout cells. Second, multiple integrins have been shown to be shuttled to the cell surface in a fibronectin-coupled manner [61, 62]. This shuttling may also regulate FN1 fibrillogenesis and assembly at the ECM [63, 64]. Therefore, we suggest that integrin trafficking, which is related to anchorage-independent growth, is affected by FN1.

Recently, c-Rel has been identified by many studies as a checkpoint regulator of tumor-promoting myeloid and Treg functions, along with fibrosis [30–32]. Our model proposes a novel benefit for targeting c-Rel in solid tumors as a stratification marker of isolation stress during metastasis. Overall, inhibiting c-Rel in solid tumors represents a potential therapeutic strategy for targeting multiple aspects of tumor biology, including tumor-promoting inflammation and metastasis.

## Methods

### Mice

The genetically engineered mouse models used in the manuscript have been previously described [37]. The mouse lines used are Ptf1a^tm1(cre)Hnak^, Kras^tm4Tyj^, Relb^tm1Fwei^, Rel^tm1Ukl^, GFP-Rel, Fn1^tm1Ref^ and Trp53^tm1Brn^ [36, 65–69]. In accordance with the European Directive 2010/63/EU, all animals are housed under specific pathogen-free conditions. Disease-free survival is generated with mice died/sacrificed due to tumor related events. The mice are monitored and scored weekly for their tumor burden. Each week mice are inspected for their weight loss, hydration status, behavior, fur and body shape, posture, excretion, breathing, pain and palpable tumor size. Mice reaching a certain burden score are sacrificed as endpoint. Mice are routinely checked for probable body recombination. Breeding is always maintained with fathers without *Ptf1a-Cre* to avoid germline recombination.

### Experimental metastasis assay via intra-tail vein injection

Littermates without *Ptf1a-Cre* from the respective genotypes between 8 and 13 weeks of age were used for tail-vein injection. The cells were trypsinized, collected as single-cell suspensions, and washed with PBS. For each mouse, 1 million cells were resuspended in 200 µL of PBS for injection. Unless a heavy burden was detected, the mice were sacrificed 28 days after injection. To analyze metastatic colonization, the pancreas, liver, lung, spleen, duodenum, kidney, lung, heart, and thymus were collected. Histopathological metastasis quantification was performed on the lungs. Two to three tissue sections per mouse were cut from the lungs at 150–200 μm intervals for H&E staining, scanning, and quantification via QuPath [70]. The metastatic colony and whole lung areas were selected via the wand tool. Their ratio was assessed as the metastatic area per field.

### In vitro cell culture

#### Cell lines

Primary mouse pancreatic cancer cells were isolated from their respective mouse tumors. Small tumor pieces are taken from fresh tumors and minced to be incubated in full culture media. The cells were routinely cultured under standard 5% CO_2_, 37 °C conditions, and tested for mycoplasma. The cell culture medium consisted of Dulbecco’s modified Eagle medium (DMEM) + GlutaMAX (#10566016; Gibco) supplemented with 10% FBS (#10082147; Gibco), NEAA (#11140050; Gibco) and 1% PenStrep (#1500–063; Gibco). The cells were used only until passage number 10. Since the GFP-Rel allele contains a hygromycin resistance cassette, GCKP and FNGCKP cells were subjected to hygromycin selection. All of the cells were validated for the recombination of their respective alleles via PCR and western blot analyses. Human pancreatic cancer cells were maintained on the basis of ATCC recommendations.

#### Spheroid culture

Adherent cells were collected as single-cell suspensions after trypsinization. All the cells were counted and serially diluted to 1000–2000 cells/mL per well for seeding on Nunclon Sphera ultralow attachment 24-well plates (Thermo Fisher Scientific 174930). The seeding number and choice of well plates are altered on the basis of the subsequent experiments. The cells were fed spheroid medium when needed. Primary spheroids were imaged 7–10 days after seeding with a brightfield microscope. Spherical forms with diameters larger than 40 μm are quantified for size with Qupath software. Primary spheroids were trypsinized, mechanically dissociated to form single-cell suspensions, and reseeded into the spheroid medium again to obtain secondary spheroids. Spheroid cultures were prepared as follows: 0.5 L of DMEM/F12 (1:1) (Gibco, 21331–020), 5 mL of Pen/Strep (Gibco, 1500–063), 2 mL of amphotericin B (R&D Systems, B23192), 5 mL of L-glutamine (Gibco, 25030–024), 10 mL of B-27 supplement (Gibco, 17504–044), and 10 µg of FGFβ (R&D Systems, 3718-FB or BioLegend, 579602).

#### Organoid formation assay with matrigel

The tumor cells were trypsinized and serially diluted to obtain multiple cell densities of 0.75, 3, 10, and 20 cells per 20 µL in Matrigel per well for 96 wp. Seven technical replicates were seeded per density. After the Matrigel solidified, 80 µL of full medium was added to each well. Seven days after seeding, each well was evaluated under a microscope for spheroid formation (yes or no). The online software ELDA, extreme limiting dilution analysis, is used to quantify stem cell frequency [71].

### RNAseq analysis

The subconfluent cancer cells (passage number 4–6) used for RNA extraction. RNA extraction is made with Maxwell^®^ 16 LEV simple RNA Purification Kit (Promega, AS1280) and the Maxwell^®^ 16 instrument (AS2000). The RNA purity, concentration, and integrity were assessed via a NanoDrop 2000 (Thermo Fisher Scientific) and gel electrophoresis. RNAs are then sent to Novogene Europe Company Limited (Cambridge, United Kingdom). Further RNA sample quality checks with bioanalyzer, mRNA library preparation with polyA enrichment, sequencing with the NovaSeq 6000 PE150 platform, and quality control of the RNA-seq data were performed by the same company.

Thirteen RNA-Seq libraries (with technical triplicates) from three conditions (5 CKP, 4 CCKP, and 4 GCKP samples) were processed via the same pipeline for compatibility. Quality control was performed via FastQC (v0.11.8) [72]. Trim Galore! (version 0.6.5) was used to trim the adapter sequences with a quality threshold of 20 [73]. The mouse genome reference was GRCm38.p6, and GENCODE release M25 was used as the transcriptome reference. The alignment was performed by using STAR aligner (v2.7.5b) [74]. Gene level read counts were obtained via Salmon (v1.2.1) for all the libraries [75]. All the samples passed the quality control requirements, with >90% of the reads uniquely mapped (>20 M uniquely mapped reads for each library) via the STAR aligner.

Differential expression analysis was performed via gene level read counts and the DESeq2 (v1.28.1) R package [76]. Genes with fewer than 5 reads across all samples were filtered as inactive genes. A gene was considered differentially expressed if the adjusted p value was less than 0.05 and if the absolute log2-fold change was greater than 1. Gene set enrichment analysis for functional analysis was performed via the clusterProfiler R package (v3.16.0) [77]. The gene sets used for functional analysis were obtained from the Molecular Signatures Database (MSigDB) [78–80]. Gene expression heatmaps show the z scores of DESeq2 VST-normalized gene-level read counts and were generated via the heatmaply R package (v1.1.0) [81].

For RNA-seq analysis of the degron cell spheroids, reads were aligned via STAR to the mm39 reference genome (settings: --quantMode GeneCounts --outSAMtype BAM SortedByCoordinate --outSAMunmapped Within --outSAMattributes Standard) [74]. A count matrix file was subsequently generated from the STAR output. Differentially expressed genes were identified via the DESeq2 package with default settings [76], with cRel-overexpressing cells used as the reference sample. Genes with FDR < 0.01 and LFC >1 or LFC < −1 were classified as differentially expressed. For visualization, BigWig files were generated with deeptools bamCoverage (settings: --binSize 1 --normalizeUsing RPKM), and tracks were displayed on the UCSC Genome Browser. Heatmaps were created via the pheatmap package.

### CUT&RUN analysis of the degron cells

A2929-cRel pAY15 stably transfected cells were seeded into 6-well plates at 400.0000 cells per well. To induce the nuclear localization of c-Rel, the cells were treated for 30 min with leptomycin-B (20 ng/mL, L2913, Sigma) in full medium. Before subsequent steps, c-Rel nuclear localization was assessed via visual GFP inspection. The required buffers were prepared, filtered through a 0.25 μm filter and stored at 4^°^C. *Buffer 1*: 5 mL of 10X eBioscience Perm/Wash Buffer, 1 tablet of complete EDTA-free protease inhibitor (Roche), 12.5 µL of 2 M spermidine (molecular grade, Sigma), and nuclease-free water up to 50 mL. *Buffer 2*: 0.05% (w/v) molecular grade saponin (25 mg in final 50 mL, Sigma), 1 tablet of complete EDTA-free protease inhibitor, 12.5 µL of 2 M spermidine, and up to 50 mL of PBS. *Antibody buffer*: 40 µL of 0.05 M EDTA, up to 10 mL of Buffer (1) *Calcium buffer*: 20 µL of 1 M CaCl_2_, up to 10 mL of Buffer (2) *2X Stop Buffer*: 200 µL of 0.5 M EDTA, 40 µL of 0.5 M EGTA, and 5 mL of Buffer 2. The entire procedure was performed on ice, and all centrifugation steps were performed at 2000 rpm for 4 min in the cold. The cells were trypsinized and collected after 3 washes with PBS to eliminate FBS-DNA on ice. The cells were incubated in 100 µL of antibody buffer for 10 min and then spun down. The cells were resuspended in 100 µL of antibody buffer with c-Rel antibody (3 µg, AF-2699 R&D Systems) and incubated overnight on ice at 4^°^C. The cells were washed 2X in 100 µL of Buffer (1) The cells were subsequently resuspended in 50 µL of Buffer 1 containing 1.5 µL of pA/G-MNase (#40366, Cell Signaling) and incubated on ice for 1 h. The samples were subsequently washed 3X in 100 µL of Buffer (2) For targeted digestion, the cells were resuspended in 50 µL of calcium buffer and incubated on ice for 30 min. Then, 50 µL of 2X Stop buffer was added to stop the reaction, and 1 µL of 0.1X Epicypher *E. coli* spike in DNA (#40366, Cell Signaling) was added. The final mixture was resuspended well, transferred to DNA low-binding eppies, incubated at 37^°^C for 15 min and spun down for 5 min at 18.500 × g. A total of 90 µL of the supernatant was carefully collected to subsequently purify the DNA according to the manufacturer’s protocol (MinElute, Qiagen). Finally, 11 µL of each sample was eluted for analysis for sample quality and sequencing by Novogene.

All sequencing data were subjected to quality checks via FastQC. The CUT&RUN reads were aligned to the mm39 reference genome via Bowtie2 (settings: --local --very sensitive) [82]. PCR duplicates and blacklisted regions were filtered out via Picard and Bedtools, respectively. Peaks were called via MACS2 (settings: -f BAMPE --call-summits --keep-dup all) [83]. Deeptools was used to generate BigWig files (settings: --binSize 1 --normalizeUsing RPGC), and genomic tracks were visualized on the UCSC Genome Browser. Heatmaps were created with deeptools bamCoverage (settings: -a 2000 -b 2000 --skipZeros --missingDataAsZero --referencePoint center) [84]. ChIPseeker was utilized for peak annotation [85].

### Immunoblot analyses

The cells and tissues were collected in RIPA buffer (150 mM NaCl, 1% NP-40, 0.5% sodium deoxycholate, 50 mM Tris, pH 8.0) supplemented with fresh protease and phosphatase inhibitor cocktail (SERVA). The lysates were incubated on ice for 20 min and sonicated three times. The supernatant after centrifugation was used for the Bradford assay (Bio-Rad, 5000006) per the manufacturer’s protocol. The lysates were diluted with 6X Laemmi buffer and denatured at 95 °C for 5 min, followed by immediate ice chill. The lysates were then run on SDS‒PAGE gels with various acrylamide concentrations depending on the target. The proteins were transferred to PVDF or NC membranes via the wet-transfer method. The membranes were incubated overnight with primary antibodies at 4 °C on a shaker, after which they were incubated with secondary antibodies for 1 h. Target visualization is performed with ECL reagents or the IR-Dye LICOR system. A list of the antibodies used for the immunoblot analyses can be found below.

**Table:** 

Target	Catalog #	Company
c-Rel	12,707	Cell Signaling
	4727	Cell Signaling
	AF-2699	R&D systems
p65/RelA	sc-372	Santa Cruz
p50/105	32360	Abcam
RelB	Sc-226	Santa Cruz
p52/100	15503-1-AP	Proteintech
IKBβ	sc-945	Santa Cruz
Zeb1	A301-922 A	Bethyl Laboratories
E-cadherin	610181	BD-Biosciences
β-Catenin	9562	Cell Signaling
HSP90	sc-7947	Santa Cruz
N-cadherin	610921	BD-Biosciences
Vimentin	8978	Abcam
β-Actin	A2228	Sigma Aldrich
P-Stat3 Y705	9131	Cell signaling
p-FAK Y397	700255	Invitrogen
FAK	66258	Proteintech
p-Src	2101	Cell signaling
Gapdh	60004	Proteintech
Fibronectin	199056	Abcam
Twist	50887	Abcam
LaminA/C	20681	Santa Cruz
IKB⍺	4814	Cell Signaling
anti-Rabbit HRP	NA934	Amersham
anti-Goat HRP	Sc-2354	Santa Cruz
anti-Rabbit IRDye 800CW	926–32211	LICOR
anti-Mouse IRDye 680RD	926–68072	LICOR

### ELISA

Blood was collected from freshly sacrificed tumor-bearing mice. The serum was fractionated with Microvette^®^ 500 Serum Gel CAT (Sarstedt, 20.1344) and snap frozen. The serum samples were diluted 1:8000 and analyzed with a Mouse Fibronectin ELISA Kit (Novus, NBP2-60517). The values are log-transformed, and a standard curve is generated via linear regression. On the basis of the absorbance, the concentration of the unknown sample was calculated.

### Tissue processing, immunohistochemistry (IHC), sirius red staining and masson’s trichrome staining

Tissues were collected and fixed in 4% PFA for 48 h. The tissues were then dehydrated and processed via a Leica ASP300S. After being embedded in paraffin, 2 μm tissue sections were rehydrated with 2 changes of Histoclear (100%−96%−70% EtOH). The slides were evaluated via H&E staining. For IHC, heat-induced antigen retrieval is performed with either citrate buffer (pH: 6) or Tris-EDTA buffer (pH: 8 or 9). Endogenous peroxidase activity was blocked with 3% H_2_O_2_. Blocking was performed for 1 h with serum according to the secondary antibody species and avidin (SP-2001 Vector). The primary antibody was diluted in a blocking solution with biotin and incubated overnight. DAB development was performed after secondary antibody and ABC-HRP incubation (Vector). The slides were then counterstained, dehydrated, mounted with Pertex, and scanned for subsequent digital pathological evaluation. For Sirius Red staining, the slides were deparaffinized, rehydrated, and stained with Weigert’s hematoxylin for 8 min and Sirius Red solution (0.5 g of Sirius Red F3B (C.I. 35782) in 0.5 L of saturated picric acid solution) for 1 h at RT. The slides were subsequently washed with 5% glacial acetic acid solution and water. For Masson’s trichrome stainings, manufacturer’s protocol is followed (Sigma-Aldrich HT15-1KT). Dehydrated and mounted slides were used for scanning. IHC quantification was performed with Qupath software [70]. With the wand tool, the whole tumor area is selected. For each staining, values for cell detection were visually inspected. Depending on the staining pattern (e.g., nuclear, diffuse cytoplasmic, speckle cytoplasmic or membrane), DAB + staining quantification was performed. The + 1, +2 and + 3 staining thresholds are automatically quantified for each slide via the ‘set intensity threshold’ command. Sirius red staining was quantified with Qupath software via a pixel classifier. From each slide region, annotations are created to generate a training image. The training image is used to train a pixel classifier. The list of antibodies used for IHC can be found below. c-Rel IHC is performed with Leica Bond RX standard protocol.

**Table:** 

Target	Catalog #	Company	Dilution	HIAR
c-Rel	AF-2699	R&D systems	1:300	Citrate pH: 6.0
CK-19	Troma III	Developmental Studies hybridoma bank	1:1500	Citrate pH: 6.0
E-cadherin	610181	BD Biosciences	1:100	Citrate pH: 6.0
β-Catenin	9562	Cell signaling	1:200	Citrate pH: 6.0
Fibronectin	199056	Abcam	1:250	Citrate pH: 6.0
CD61/Integrin-β3	NBP2-67416	Novus	1:200	Tris-EDTA pH: 9.0
Vimentin	5741	Cell signaling	1:500	Citrate pH: 6.0

### Flow cytometry analyses

Cultured cells under adherent or spheroid culture conditions were trypsinized and suspended as single cells in FC buffer (PBS, 1% FBS, and 0.5 mM EDTA). Then, the cells were incubated for 30 min–1 h with fluorophore-conjugated antibodies to detect CSC surface expression markers. For compensation or unmixing, single strains of each antibody are used. The cells were finally resuspended in either 300 µL of PBS with 1:5000 Zombie Red (BioLegend 423110) or 300 µL of FC buffer with 1:5000 DAPI (Cell Signaling, 4083 S), depending on the panel. Before analysis, the suspensions were passed through a 35 μm strainer (Corning 352235). Flow runs were performed with a Gallios flow cytometer (Beckman Coulter), an Attune flow cytometer (Thermo Fisher Scientific), or a Cytek Aurora flow cytometer. Downstream analyses were performed further with FlowJo software. The list of antibodies used for the flow cytometry analyses can be found below.

**Table:** 

Flow Cytometry			
Target	Catalog #	Company	Conjugate
E-cadherin	147308	BioLegend	AlexaFluor 647
Cxcr4	130-118−682	Miltenyi	PE
EpCAM	130-117−866	Miltenyi	PE-Vio770
Sca1	108142	BioLegend	AlexaFluor 700
CD133	141216	BioLegend	AlexaFluor 647
CD44	103019	BioLegend	Pacific Blue
CD61/Integrin-β3	130-122−151	Miltenyi	PE-Vio770

### RT‒qPCR

RNA was isolated as described in the RNAseq section. A total of 600 ng of RNA was used for cDNA synthesis with a GoScript reverse transcriptase kit (A2791, Promega) according to the manufacturer’s protocol. The cDNA was diluted 1:5, and 5 µL was added to each PCR mixture via Go-Taq qPCR Master Mix (Promega). RT‒qPCR was performed with either a LightCycler 480 (Roche) or a qTOWER^3^ G Cycler (Analytic Jena). The specificity of each primer pair was tested via gel electrophoresis and Tm calling. A list of primers and their sequences can be found below.

**Table:** 

RT‒qPCR primers		
Target	fwd	rev
mFn1	GGTGACACTTATGAGCGCCCTA	AACATGTAGCCACCAGTCTCAT
mEDA_Fn1	TGACCAACATTGATCGCCCT	GATTCCATCCTCAGGGCTCG
mEDB_Fn1	ATACCGTTGTCCCAGAGGTG	GGAAGAGTTTAGCGGGGTCC
mItgb3	ATGCGAACGCGGCGGCCG	TTAAGTGCCCCGGTAGGTGATATTGGTGA
mItgav	ATGGCTGCTCCCGGGCGC	TCAGGTTTCAGAGTTTCCT
mItga5	CCGTGGACTTCTTCGAGCC	CTGTTGAATCAAACTCAATGGGC
mRel	AGACTGCGACCTCAATGTGG	GCACGGTTGTCATAAATTGGGTT
mCdh1	AGGTTTTCGGGCACCACTTA	TGATGTTGCTCTCCCCAAGT
mZeb1	CCACTGTGGAGGACCAGAAT	CTCGTGAGGCCTCTTACCTG
mZeb2	TAGCCGGTCCAGAAGAAATG	GGCCATCTCTTTCCTCCAGT
mSnai1	GGTCCCCAACTACGGGAAAC	CTGTAGGGGCTCACTGGGATT
mSnai2	TGGTCAAGAAACATTTCAACGCC	GGTGAGGATCTCTGGTTTTGGTA

### Analysis of the transcriptomics dataset

#### Human PDAC RNAseq_transcriptomic analyses

The Bailey, Jandaghi, Janky, and TCGA datasets were used for GSEA [86–89]. The samples are ranked on the basis of *REL* expression and divided into two groups from the median as LOW vs. HIGH. The KEGG and HALLMARK gene sets were used to perform GSEA via GSEA_4.3.2 [78–80, 90].

For the correlation of *REL* vs. *FN1/ITGB3/SNAI1/SNAI2/TGFB1/ZEB2* mRNAs and the survival curves for *FN1,* *ITGB3, ITGA5,* and *ITGAV* in the TCGA_PAAD dataset, the UCSC_Xena online tool was used [91]. Only the primary tumor samples were subsequently analyzed.

#### pdacR stromal subtyping

The analyses were performed via the online tool pdacR (http://pdacr.bmi.stonybrook.edu/) [34]. The datasets are filtered as follows: TCGA-only whitelisted samples [89], Puleo-no prefiltering [92], CPTAC3-cellularity_call_from_VAF: Acceptable, sample_type: Primary Tumor [93]. The gene sets used for selection are as follows: Moffit Normal 25, Activated 25, F13_NormalStroma top100–top250, and F5_ActivatedStroma top100–top250. As a sample track, expression signature 1-user selected genes 1 is used, which is REL. The sample and gene methods used were Pearson correlation.

#### scRNA-seq data analysis

Previously published scRNA-Seq datasets, GSE205013 [94] and GSE154778 [95], were retrieved from the NCBI GEO database. Barcodes, features, and matrix files were downloaded and imported into Python 3.9 via the Scanpy package (version 1.9.3) [96]. Initial quality control was performed on each sample individually following best practices [97]. Briefly, low-quality cells with a mitochondrial gene content < 15% were filtered out, along with genes present in fewer than 10 or 20 cells. Doublets were subsequently removed via the R package scDblFinder [98] with default settings. The datasets were then concatenated, and the cells were further filtered on the basis of the following criteria: fewer than 200 genes, fewer than 1500 counts per cell, or more than 150,000 counts per cell. Additionally, cells with >1% of transcripts representing erythroid genes were excluded. The data were normalized via the shifted logarithm method and log1p transformation. Dimensionality reduction was carried out on scaled data by computing PCA and a neighborhood graph. Batch correction and integration were performed via batch-balanced k-nearest neighbors (bbknn) (version 1.6.0) [99]. Uniform manifold approximation and projection (UMAP) [100] was applied for two-dimensional visualization, and cells were clustered via the Leiden algorithm [101] with the default setting. Cell types were annotated via Scanpy’s rank_genes_groups function with default settings, method=’wilcoxon’, and corr_method=’bonferroni’. For downstream analysis, epithelial clusters were subsets, and cells were stratified on the basis of REL expression levels using a predefined threshold of 0.1, above and below which cells were classified as RELHigh vs. RELLow, respectively.

### Auxin inducible degron 2 system (AID2)

pAY15 (OsTIR1(F74G) mAID-EGFP-NLS) was a gift from Masato Kanemaki (Addgene plasmid # 140534; http://n2t.net/addgene:140534; RRID: Addgene_140534) [42]. To clone the mouse *Rel* gene, PCR was conducted with cDNA generated from a GCKP cancer cell. The product and the vector are restriction digested by BamHI and MfeI, followed by ligation and transformation. The selected final clone was sequenced and validated. CCKP cells were transfected with either pAY15 (empty vector) or Rel-pAY15. Transient transfection was not suitable for the degradation of c-Rel protein after 5phIAA treatment (varying doses and time points, 7392, Tocris). On the basis of personal communication with Dr. Kanemaki, the pAY15 and Rel-pAY15 constructs are genome integrated via transposase expression. Briefly, pCMV-TOL2 and pAY15 constructs are cotransfected into CCKP cells via polyplus JET Optimus, followed by puromycin selection (the concentration is selected on the basis of titration for each cell line). pCMV-Tol2 was a gift from Stephen Ekker (Addgene plasmid #31823; http://n2t.net/addgene:31823; RRID: Addgene_31823.

### Immunocytochemistry

The cells were seeded in an 8-well chamber at 40,000 cells/well (94.6140.802, ×well, Sarstedt). After treatment, the cells were washed with HBSS (Mg/Ca ++) and fixed at 37 °C for 10 min with 4% PFA in HBSS. After 3 washes with HBSS, the cells were permeabilized with 0.1% Triton-X in PBS for 5–7 min at RT. At 4 °C, the cells were incubated with primary antibody diluted in 2% filtered BSA. The next day, after washing, the cells were incubated with the appropriate secondary antibodies for 1 h at RT. After the final washes, the cells were mounted with DAPI (H-1200–10, Vector) and visualized with a Leica DMi8 microscope. For the staining, the following antibodies were used: E-cadherin (BD Biosciences, 610181) and c-Rel (Santa Cruz, Sc-71).

### Collagen contraction assay

Rat tail collagen type I (9007-34−5, Corning) was diluted to 3 mg/mL in sterile 0.1% acetic acid to prepare a working solution. For each cell line, 200,000 cells were prepared in 1 mL of medium and mixed with 0.5 mL of collagen working solution. For solidification of the collagen/cell culture mixture, the NaOH amount was titrated. After NaOH addition, the solution was immediately aliquoted as 3 × 500 µL technical replicates into 24-well plates. Each gel was carefully dissociated from the surrounding wall with a 10 µL pipette tip after the gel solidified. Gel contraction was observed for up to a few days until a difference was observed. The plates were scanned, and the gel surface areas were quantified via ImageJ software.

### Fibronectin adhesion assay

Human fibronectin (1918-FN R&D Systems) was diluted to a concentration of 1 µg/mL in PBS supplemented with 0.5 mM Mg^++^ and 0.9 mM Ca^++^ and aliquoted as 100 µL per well into a 96wp black/clear bottom (165305, Thermo Fisher Scientific). The plates were incubated overnight at 4 °C and then blocked with 1% BSA-fraction V in PBS for 30 min at RT. After the plates were slightly dried, 100,000 cells in 100 µl of PBS (supplemented with Mg^++^ Ca^++^) were seeded on the wells in triplicate. The cells were spun down for 2 min at 300xrcf with a slow break. After 4 h, the unattached cells were removed by washing with 2X PBS. To normalize the number of seeded cells, another group of cells was incubated for 24 h without serum, allowing all the seeded cells to attach. The relative cell number was assessed via a CyQuant viability assay (C7026, Thermo Fisher Scientific) per the manufacturer’s protocols.

### Drug treatment and cell viability assay

Each cell line was seeded at 10,000 cells per well in triplicate per dose in 96-well plates. The next day, once the cells were attached, treatment started. Both dasatinib (HY-10181) and defactinib (HY-12289) were purchased from MedChemExpress as 10 mM stock solutions in DMSO and were serially diluted to final concentrations. The cells were incubated with 100 µL of medium containing drugs for 48 h. Viability was quantified via CellTiter-Glo^®^ 2.0 Cell Viability reagent (G9241, Promega) according to the manufacturer’s protocols. Viability was normalized to that of the no-treatment control for each cell line.

### Co-immunoprecipitation

Fifty million cells were washed with PBS and lysed with 1 mL of Co-IP lysis buffer (1% Triton-X, 150 mM NaCl, 2 mM EDTA, 50 mM Tris-Cl pH 8, fresh protease/phosphatase inhibitors, fresh 0.5 mM DTT, fresh 100 µM PMSF) for 30 min on ice. The lysed sample was scraped and collected in a 1.5 mL Eppendorf tube, sonicated and passed through a syringe a few times. The lysate was centrifuged for 15 min at 4 °C at maximum speed. All of the remaining centrifugation steps were performed at 2500 × g and 4 °C for 3 min unless otherwise indicated. The lysate was precleared for binding to protein A/G microbeads (20241, Pierce Protein A/G Agarose, Thermo Scientific), 20µL beads were added, and the volume was adjusted to 1 mL. 10% of the lysate is set aside as input. The remaining lysate was split into 4 parts for incubation with primary antibodies at 4 °C on a rotator. One to two milligrams of protein per cell was incubated with 2 µg of antibody at 4 °C on a rotator for 2 h. The antibodies used were anti-c-Rel sc-71, anti-p65 sc-372, anti-RelB sc-226, and rabbit IgG (sc-2027) from Santa Cruz. The beads were equilibrated with wash buffer and blocked with 1% BSA for 1 h in lysis buffer. The mixture was mixed with blocked beads and incubated on a rotator at 4 °C overnight. After centrifugation, the supernatant was collected as a flow-through for quality assessment. The precipitated beads were washed 2x with 500 µL of lysis buffer and 1 mL of 0.1% PBS-Triton X-100. Finally, the beads were suspended in 100 µL of lysis buffer + 20 µL of Laemmi buffer, boiled and centrifuged for further use in western blotting. The supernatant was used for gel loading.

### Nuclear fractionation

The buffers used for fractionation were as follows: hypotonic buffer (10 mM HEPES, 1.5 mM MgCl_2_, 10 mM KCl, 0.5 mM DTT, 0.1% NP40 and freshly added protease/phosphatase inhibitors) and nuclear buffer (20 mM HEPES, 100 mM KCl, 100 mM NaCl, 0.1% NP40, 0.5 mM DTT and freshly added protease/phosphatase inhibitors). Adherent cells grown to subconfluency in 10 cm plates were washed with cold PBS and lysed with cold 1 mL hypotonic buffer. The buffer was added dropwise to the plates, and the plates were incubated on ice for 30 min and subsequently collected by scraping. The cytoplasm was collected by centrifugation at 13,200 rpm for 15 min at 4 °C. The supernatant was the cytoplasmic fraction. The nuclear pellet was washed 5x with 300µL of hypotonic buffer. Finally, the nuclear pellet was resuspended in 200µL of nuclear buffer. The fraction was passed through a 31G syringe until smooth and incubated with 0.5 µL of benzonase (9025-65−4, Sigma) for 20 min on ice. The lysate was centrifuged for a maximum of 15 min, and the supernatant was denatured by boiling for 5 min at 95 °C with Laemmi buffer.

### Preparation of patient TMAs and their IHC results

All patients who underwent duodenopancreatectomy from 2007 to 2013 at the Hepatobiliary and Pancreatic Surgery Unit (General and Digestive Tract Surgery Department, Clinico San Carlos Hospital) were initially assessed for eligibility, and samples were provided by its institutional biobank (B.0000725; PT17/0015/0040; ISCIII-FEDER). The institutional review board (IRB) of University Hospital Clinico San Carlos evaluated the present study, granting approval on 10 March 2017 with approval number n° 17/091-E. The main criteria for eligibility were patients with a final histopathological diagnosis of localized pancreatic ductal adenocarcinoma (PDAC) who underwent surgery. Tumors were surgically resected and were formalin fixed and paraffin embedded immediately for pathologic diagnosis. To assess survival, only patients with a confirmed pathological diagnosis of PDAC and available data for progression-free and overall survival were included in the study.

#### Tissue microarray

Tissue microarrays were constructed with 190 patient samples, 2 cores per patient, via the MTA-1 tissue arrayer (Beecher Instruments, Sun Prairie, WI, USA), with available formalin-fixed and paraffin-embedded tissues. A hollow needle was used to obtain a tissue core 1 mm in diameter from selected tumor regions in formalin-fixed and paraffin-embedded tissues. These tissue cores were then inserted into a recipient paraffin block with precise spacing, resembling an array pattern. Sections from this paraffin block were then cut with a microtome and mounted on a microscope slide for evaluation via immunohistochemistry.

#### Immunohistochemistry

Staining was conducted in 2 μm sections. First, the slides were deparaffinized by incubation at 62 °C for 10 min and incubated for antigen retrieval via PT-Link (Dako, Glostrup, Denmark) for 20 min at 97 °C in a low-pH buffered solution (EnVision Dako kit). The samples were then incubated with peroxide (EnVision Flex peroxidase blocking reagent) to block endogenous peroxidase activity. The biopsies were stained overnight with diluted antibodies against c-Rel (1:50; R&D Systems AF-2699) followed by incubation with the appropriate anti-Ig conjugated with horseradish peroxidase (Anti-mouse/Anti-rabbit EnVision FLEX-HRP Labeled Polymer; Dako, Glostrup, Denmark) for 20 min. Visualization of the sections was carried out with 3,3′-diaminobenzidine (DAB, Dako, Glostrup, Denmark) as a chromogen for 5 min. Hematoxylin (Harris’ Hematoxylin, Sigma‒Aldrich, San Luis, MO, USA) was used for counterstaining for 2 min.

All the antibodies and anti-Ig horseradish peroxidase-conjugated antibodies presented high specificity, and no positive results were obtained from these antibodies individually. To determine the best immunohistochemistry conditions and maximize the specificity of the antibodies, different healthy control tissues were used as positive controls according to “*The Human Protein Atlas*” (http://www.proteinatlas.org (accessed on 12 July 2022)). The evaluation of the staining was performed by an expert pathologist (M.J.F.-A.).

#### Quantification of immunohistochemistry

The evaluation of the immunoreactivity of the different antibodies via immunohistochemistry was carried out via a semiquantitative method called HistoScore, which considers the intensity of staining and the percentage of positively stained cells. The quantification is made based on cytoplasmic expression. The HistoScore (Hscore) ranged from 0 to 300 and was multiplied by the percentage of positively stained cells for each low, medium, or high staining intensity. The final score was determined via the following formula:

$$\mathrm H-\mathrm{Score}=\left(1\times\%\mathrm{Low}\right)+\left(2\times\%\mathrm{Medium}\right)+\left(3\times\%\mathrm{High}\right)$$

### Multiplex Immunofluorescence

FFPE tissue sections were stained using the Opal 6-Plex Manual Detection Kit for Whole Slide Imaging (Akoya Biosciences, SKU NEL861001KT) with antigen retrieval in Tris-EDTA buffer (pH 9.0). Whole-slide images were acquired on the Akoya PhenoImager HT across DAPI, Opal 480, 520, 570, 620, 690, and 780 channels. Spectral unmixing was performed via inForm analysis software. Unmixed tiles were exported and reconstructed by tile stitching in QuPath (v0.6, arm64). All downstream analyses were performed in QuPath (v0.5, arm64). The list of antibodies and their staining protocol is given below in the table.

**Table:** 

Marker	Antibody dilution	AR	Company	Ref.No.	Clone	OPAL (dilution)
CD61/Integrin β3	1:300	pH9, Tris-EDTA	Novus	NBP2-67416	SJ19-09, mRb	480 (1:300)
PanCK	1:1000	pH9, Tris-EDTA	Proteintech	26411-1-AP	Polyclonal, Rb	520 (1:250)
GFP	1:2500	pH9, Tris-EDTA	Biosynth	20R-GR011	Polyclonal, Rb	520 (1:250)
SOX9	1:300	pH9, Tris-EDTA	Millipore	AB5535	Polyclonal, Rb	570 (1:200)
Fibronectin	1:1000	pH9, Tris-EDTA	abcam	ab199056	EPR19241-46, mRb	620 (1:200)
Vimentin	1:500	pH9, Tris-EDTA	Cell Signaling	#5741	D21H3, mRb	690 (1:200)
Ki67	1:2500	pH9, Tris-EDTA	abcam	ab15580		780 (1:30)
Podoplanin	1:250	pH9, Tris-EDTA	Novus	NBP1-90211	Polyclonal, Rb	690 (1:200)

### Statistics

Prism 9 software (GraphPad Software, Inc.) was used for statistical analysis. The p values used for all the statistics are as follows: ns *p* > 0.05, *p ≤ 0.05, **p ≤ 0.01, ***p ≤ 0.001, ****p ≤ 0.0001. The data are displayed as the means ± standard errors of the means (SEMs).

## Supplementary Information

Supplementary Material 1.Supplementary Material 1. Supplementary Figure 1. A) Immunoblot analyses of c-Rel and β-actin expression in human and mouse cancer cells isolated from primary PDAC tumors that spontaneously formed in GEMMs. Each mouse line was isolated from an individual tumor. B) Representative IHC images indicating c-Rel expression in mouse PDAC primary (CKP n= 18, CK n=5, CKPhet n=9) and metastatic tumors (liver_met n= 4, lung_met n=3) and the histogram graph for the percentage of c-Rel expressing cancer cells. C) The given PDAC patient datasets were analyzed to determine the prognostic impact of REL expression on overall survival (OS). The table is generated with data from pdacR analysis. D) c-Rel H-scores are compared for patients stratified based on various clinical parameters. E) Representative images for c-Rel IHC in CCKP and GCKP tumors.

Supplementary Material 2. Supplementary Figure 2. A) RT‒qPCR analysis of the given targets with cDNA synthesized from bulk tumor tissue RNA. One-way ANOVA was used for statistical analyses with p values; Rel p<0.0001, Cdh1 p=0.0139, Zeb1 p=0.0096, Zeb2 p=0.0031, Snai1 p=0.0163, Snai2 p=0.0011. Tukey’s multiple comparison test results are displayed in the figure. B) Representative brightfield images of CKP and CCKP cells (biological replicates, n=4 per genotype) treated with TGFβ. The cells were analyzed for surface E-cadherin expression via flow cytometry after two days of induction. The relative mean fluorescence intensity (MFI) was compared via two-way ANOVA (genotype factor p=0.0152. Šídák's multiple comparison tests between genotypes are shown in the figure. C) Relationship of REL with various EMT-related signatures in PDAC patient datasets and their statistics. The graphs are adapted from the EMTome database. The list of the signatures is given as a table. D) Multiple human PDAC RNAseq datasets were divided from the median based on REL expression and analyzed for the HALLMARK_EMT GSEA signature. E) CCKP, CKP and GCKP cells (n=4 biological replicates per genotype) were treated with dasatinib (Src-i) or defactinib (FAK-i) and analyzed for relative viability (Two-way ANOVA, the genotype factor, dasatinib p= 0.0537, defactinib p= 0.1454). Tukey’s multiple comparison test results for each dose are displayed in the figure.

Supplementary Material 3. Supplementary Figure 3. A) Representative fluorescence images of c-Rel nuclear localization in CKP cells after TNF-α and leptomycin-B treatment for 20 minutes. B) Co-immunoprecipitation analysis of RelA, RelB, c-Rel and rabbit IgG as negative controls. Ft: flow through, Ip: immunoprecipitate. The antibodies used for immunoprecipitation (IP) are listed above, whereas the antibodies used for immunoblotting are listed on the left. C) Total cell lysates were immunoblotted for the expression of multiple NF-κB proteins. Each cell line was isolated from a separate mouse autochthonous tumor (mPDAC) with the CK or CKA (p65/RelA knockout) genotype. The CKA model expresses an NLS-truncated RelA protein. Signal is normalized to β-actin. On the graph t-test p-values are given. D) Immunoblot analyses of the given NF-κB proteins in cell lysates obtained from CCKP, CKP and GCKP cells cultured under basal conditions. Signal is normalized to β-actin and plotted (p-values: RelA, RelB, p50, p100 n.s., p105 p=0.0025, p52 p=0.0214, Ikbα p=0.0138, Ikbβ p=0.0052) E) Nuclear fractionation was performed with cell lines isolated from CK, CKA, CKP and CKPA tumors (n= 2 per genotype) and immunoblotted for the given proteins. Nuclear signal is normalized to Lamin A/C for quantification. F) Nuclear fractions were isolated from CCKP, CKP and GCKP cells and immunoblotted for the given NF-κB proteins. Signal is normalized to Lamin A/C (p-values: RelA p=0.0129, RelB p=0.0019, p50 p=0.0235, p52 n.s.) G) With pdacR, multiple human PDAC RNA-seq datasets are analyzed for REL mRNA correlation to the Moffitt normal vs activated stroma top 25–100 and 250 gene signatures. The top correlation graph represents data from the TCGA cohort. The Pearson-R values and their p values are given in the table below. H) A human PDAC proteomics dataset was analyzed for c-Rel and FN1 expression in tumor cells or stroma in samples stratified on the basis of their sub-TME types. A one-way ANOVA test for a linear trend is displayed in the figure. I) Tumor cell c-Rel protein expression vs FN1 protein expression in the tumor stroma correlation plot. J) Selected GSEA results for the GCKP vs CKP and GCKP vs CCKP pairs on the basis of RNA-seq analyses. K) Human PDAC RNA-seq datasets were analyzed for KEGG-ECM receptor interaction pathways. The samples were divided into medians on the basis of REL expression. L) Two human PDAC scRNA-seq datasets were analyzed for REL expression in multiple given cell types. UMAPs showing REL levels in various cell types. The cell types are determined on the basis of the expression matrix on the left. Violin plots showing the relative REL values in the given cell types. Dot plots at the bottom display integrin expression levels and the fraction of cells expressing them in the epithelial cell compartment. Epithelial cells are divided into HIGH and LOW groups on the basis of REL expression.

Supplementary Material 4. Supplementary Figure 4. A) REL mRNA fpkm values were compared with paired t tests from an RNA-seq experiment performed with human PDAC cell lines cultured under adherent and nonadherent conditions. B) Western blot analyses of patient cell lines cultured under adherent and nonadherent conditions. The data were normalized to the total protein signal. C) Surface expression of multiple CSC markers was assessed via flow cytometry. For each genotype, 6 separate lines were used. The cells were cultured under standard adherent conditions. One-way ANOVA with multiple comparison test results is given in the figure. D) A schematic demonstrating the auxin-inducible degree 2 (AID2) system. A degron (mAID, miniAID)-tagged c-Rel recruits OsTIR (F74G mutant), which facilitates E3 ligase complex formation in the vicinity after auxin analog (5phIAA) treatment. Subsequent protein ubiquitination leads to proteasome-mediated protein degradation in a short time. E) Green fluorescence images of stably transfected CCKP cells. While the empty vector produced GFP mostly in the nucleus, c-Rel tagged with GFP was present mostly in the cytoplasm. F) Flow cytometry histogram graphs of the GFP signal in degron cells indicating that 5phIAA induced a reduction in the GFP signal after 2 hours. Notably, CCKP cells already have a basal EGFP signal due to the recombined c-Rel flox allele. A time-dependent reduction in GFP-tagged c-Rel expression upon 5phIAA treatment is shown in the immunoblot below. G) Representative microscopy images of CCKP cells stably transfected with either empty vector (pAY15) or c-Rel (Rel-pAY15) and treated with 5 ng/mL TGFβ for two days. The relative surface expression of E-cadherin was analyzed via flow cytometry (c-Rel factor p=0.0078). H) Representative microscopy images of degron cell spheroids. The left and right panels show A2929 and A2770 CCKP cells, respectively, which were stably transfected. To rescue c-Rel expression from high to low, the auxin analog 5phIAA is given at multiple doses. The average spheroid diameter was quantified and analyzed (genotype X auxin factors A2929 p= 0.0009 and A2770 p=0.0007). I) Surface expression of CD61 was assessed via flow cytometry analysis of spheroids (genotype X auxin factors, A2929 p=0.0017 and A2770 p=0.0008). J) Flow cytometry analysis of the surface expression of EpCAM and CD133 in degron cells with or without 5phIAA treatment. Each dot represents one of the CCKP cell lines. Two-way ANOVA indicated that the variation factors were not significant. K) A multiplex IF image from CCKP, CKP and GCKP tumors are split to channels.

Supplementary Material 5. Supplementary Figure 5. A) CUT&RUN and RNAseq tracks displaying the expression levels of the Snai1, Snai2, Tgfb1, Zeb2, Itga5 and Itgav genes in mock-transfected (pAY15), c-Rel-overexpressing (Rel-pAY15), and degron-induced c-Rel-overexpressing A2929 (Rel-pAY15 + 5phIAA) cell lines. B) Pearson correlations of REL with SNAI1, SNAI2, TGFB1, ZEB2, ITGA% and ITGAV mRNAs in the TCGA cohort. The graphs are adapted from the UCSC-Xena database. C) Kaplan-Meier survival curves for ITGA5 and ITGAV expression in the TCGA cohort. Samples are divided from median expression. OS: overall survival, n= 178; DSS: disease-specific survival, n=172; DFI: disease-free interval, n=69; PFI: progression-free interval, n=178. The graphs are adapted with data from the UCSC-Xena TCGA PAAD dataset. D) Correlation of c-Rel expression with Itgβ3, cancer cell FN1 and stromal FN1 expression in mouse CKP tumors. Representative staining images from the TMAs are given. E) Correlation of c-Rel versus ITGA5 H-scores in patient TMAs on the left plot. On the right, overall survival curve for the ITGA5 H-score given. F) Average patient TMA H-scores are analyzed in samples divided based on various clinical parameters. G) Heatmap displaying DEGs whose promoters are also occupied by c-Rel in degron cells (up- or downregulated). H) Overrepresentation analysis performed by Enrichr for Rel-pAY15 cells. The analysis is performed only with the genes that are differentially expressed and downregulated in Rel-pAY15 cells compared with those in pAY15 only or Rel-pAY15 cells induced with 5phIAA. From each set, only the top 5 hits are selected on the basis of their combined scores.

Supplementary Material 6. Supplementary Figure 6. A) RT‒qPCR analysis of the given targets with cDNA synthesized from bulk tumor tissue RNA. One-way ANOVA was used for statistical analyses with p values; Rel p<0.0001, Fn1, p<0.0001, EDA p=0.001, and EDB p=0.0041). B) Bulk tumor tissue lysates were immunoblotted for FN1, and HSP90 was used as a loading control. The relative band intensity quantifications are given below. For statistical analysis, a t test was used. C) Representative FN1 IHC images of the given primary tumor sections and their quantification. The close-up images below indicate the absence of FN1 in cancer cells (One-way ANOVA p=0.0001). D) Representative CD61 IHC images of the given primary tumor sections and their quantification (One-way ANOVA p= 0.0091). E) FN1 and CD61 IHC images of the lung colonies formed after IV injection. (One-way ANOVA, FN1 p= 0.008, CD61 p=0.0144). For all of the one-way ANOVA test graphs, Tukey’s multiple comparison test results are displayed in the figure.

Supplementary Material 7. Supplementary Figure 7. Uncropped immunoblot images used in the entire manuscript.

## Data Availability

The datasets generated and analysed during the current study are available in the GEO repository, [https://www.ncbi.nlm.nih.gov/geo/query/acc.cgi?&acc=GSE291806, https://www.ncbi.nlm.nih.gov/geo/query/acc.cgi?&acc=GSE291961, https://www.ncbi.nlm.nih.gov/geo/query/acc.cgi?&acc=GSE291889]. The accession number IDs are as given: (1) CCKP-CKP-GCKP cancer cell RNAseq related to Fig. 3F-H-I and Supp. Figure 3 F, GSE291806; (2) Stably transfected degron cell in spheroid cultures, RNAseq related to Fig. 5A-C and Supp. Figure 5A-C-D, GSE291961; (3) Stably transfected degron cell A2929 Cut&Run related to Fig. 5 A, Supp. Figure 5A-C, GSE291889. The accessions are currently private, but could be released upon request.
