# Supplementary figures and images for: c-Rel drives pancreatic cancer metastasis through fibronectin-integrin signaling-induced isolation stress resistance and EMT
Source: Mol Cancer. 2025 Dec 15;25:16. doi: 10.1186/s12943-025-02486-5 (PMC12849452; doi:10.1186/s12943-025-02486-5)

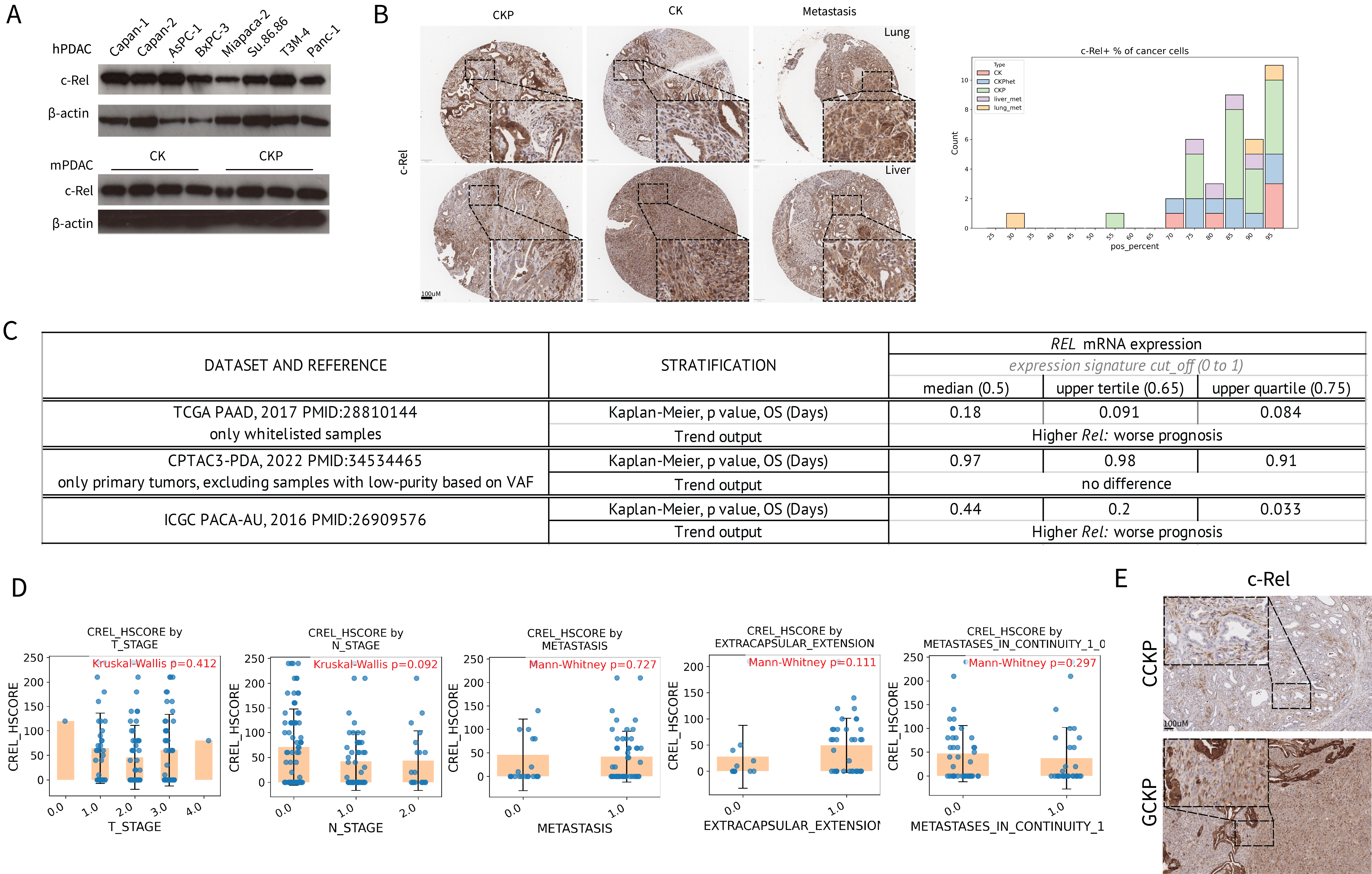

Supplement: Supplementary file 1 — Supplementary Material 1.Supplementary Material 1. Supplementary Figure 1. A) Immunoblot analyses of c-Rel and β-actin expression in human and mouse cancer cells isolated from primary PDAC tumors that spontaneously formed in GEMMs. Each mouse line was isolated from an individual tumor. B) Representative IHC images indicating c-Rel expression in mouse PDAC primary (CKP n= 18, CK n=5, CKPhet n=9) and metastatic tumors (liver_met n= 4, lung_met n=3) and the histogram graph for the percentage of c-Rel expressing cancer cells. C) The given PDAC patient datasets were analyzed to determine the prognostic impact of REL expression on overall survival (OS). The table is generated with data from pdacR analysis. D) c-Rel H-scores are compared for patients stratified based on various clinical parameters. E) Representative images for c-Rel IHC in CCKP and GCKP tumors. [file 12943_2025_2486_MOESM1_ESM.png]

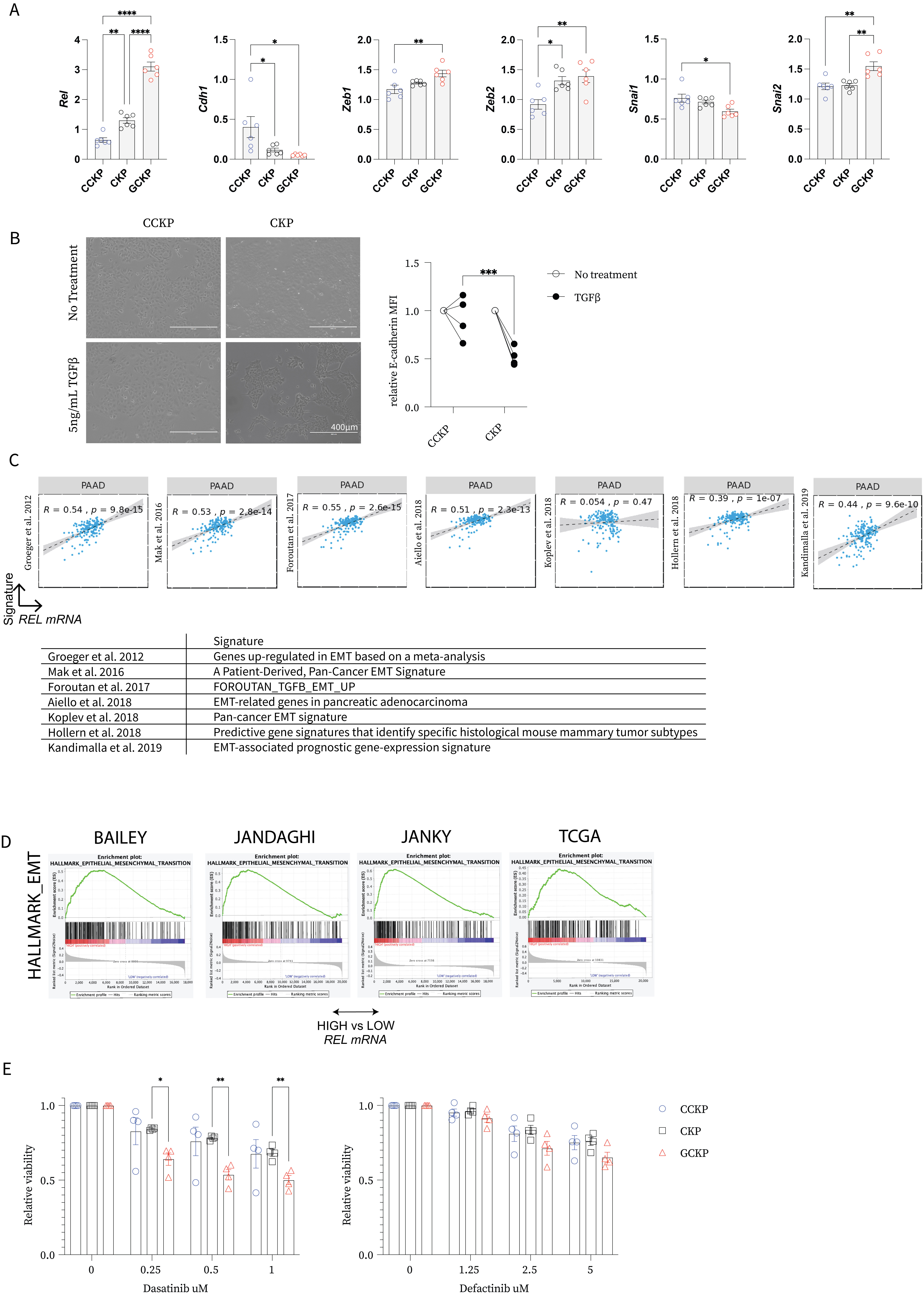

Supplement: Supplementary file 2 — Supplementary Material 2. Supplementary Figure 2. A) RT‒qPCR analysis of the given targets with cDNA synthesized from bulk tumor tissue RNA. One-way ANOVA was used for statistical analyses with p values; Rel p<0.0001, Cdh1 p=0.0139, Zeb1 p=0.0096, Zeb2 p=0.0031, Snai1 p=0.0163, Snai2 p=0.0011. Tukey’s multiple comparison test results are displayed in the figure. B) Representative brightfield images of CKP and CCKP cells (biological replicates, n=4 per genotype) treated with TGFβ. The cells were analyzed for surface E-cadherin expression via flow cytometry after two days of induction. The relative mean fluorescence intensity (MFI) was compared via two-way ANOVA (genotype factor p=0.0152. Šídák's multiple comparison tests between genotypes are shown in the figure. C) Relationship of REL with various EMT-related signatures in PDAC patient datasets and their statistics. The graphs are adapted from the EMTome database. The list of the signatures is given as a table. D) Multiple human PDAC RNAseq datasets were divided from the median based on REL expression and analyzed for the HALLMARK_EMT GSEA signature. E) CCKP, CKP and GCKP cells (n=4 biological replicates per genotype) were treated with dasatinib (Src-i) or defactinib (FAK-i) and analyzed for relative viability (Two-way ANOVA, the genotype factor, dasatinib p= 0.0537, defactinib p= 0.1454). Tukey’s multiple comparison test results for each dose are displayed in the figure. [file 12943_2025_2486_MOESM2_ESM.png]

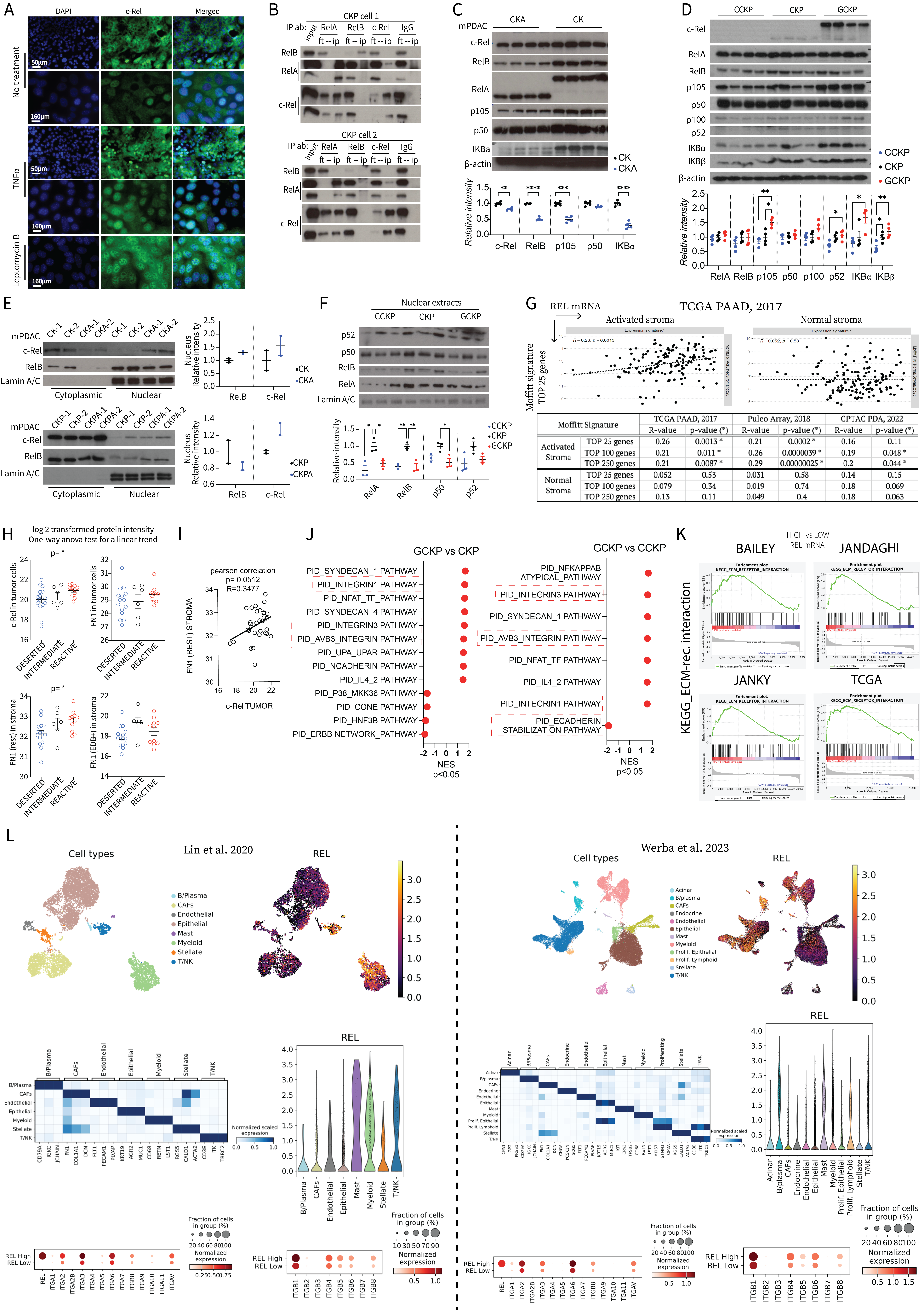

Supplement: Supplementary file 3 — Supplementary Material 3. Supplementary Figure 3. A) Representative fluorescence images of c-Rel nuclear localization in CKP cells after TNF-α and leptomycin-B treatment for 20 minutes. B) Co-immunoprecipitation analysis of RelA, RelB, c-Rel and rabbit IgG as negative controls. Ft: flow through, Ip: immunoprecipitate. The antibodies used for immunoprecipitation (IP) are listed above, whereas the antibodies used for immunoblotting are listed on the left. C) Total cell lysates were immunoblotted for the expression of multiple NF-κB proteins. Each cell line was isolated from a separate mouse autochthonous tumor (mPDAC) with the CK or CKA (p65/RelA knockout) genotype. The CKA model expresses an NLS-truncated RelA protein. Signal is normalized to β-actin. On the graph t-test p-values are given. D) Immunoblot analyses of the given NF-κB proteins in cell lysates obtained from CCKP, CKP and GCKP cells cultured under basal conditions. Signal is normalized to β-actin and plotted (p-values: RelA, RelB, p50, p100 n.s., p105 p=0.0025, p52 p=0.0214, Ikbα p=0.0138, Ikbβ p=0.0052) E) Nuclear fractionation was performed with cell lines isolated from CK, CKA, CKP and CKPA tumors (n= 2 per genotype) and immunoblotted for the given proteins. Nuclear signal is normalized to Lamin A/C for quantification. F) Nuclear fractions were isolated from CCKP, CKP and GCKP cells and immunoblotted for the given NF-κB proteins. Signal is normalized to Lamin A/C (p-values: RelA p=0.0129, RelB p=0.0019, p50 p=0.0235, p52 n.s.) G) With pdacR, multiple human PDAC RNA-seq datasets are analyzed for REL mRNA correlation to the Moffitt normal vs activated stroma top 25–100 and 250 gene signatures. The top correlation graph represents data from the TCGA cohort. The Pearson-R values and their p values are given in the table below. H) A human PDAC proteomics dataset was analyzed for c-Rel and FN1 expression in tumor cells or stroma in samples stratified on the basis of their sub-TME types. A one-way ANOVA test [file 12943_2025_2486_MOESM3_ESM.png]

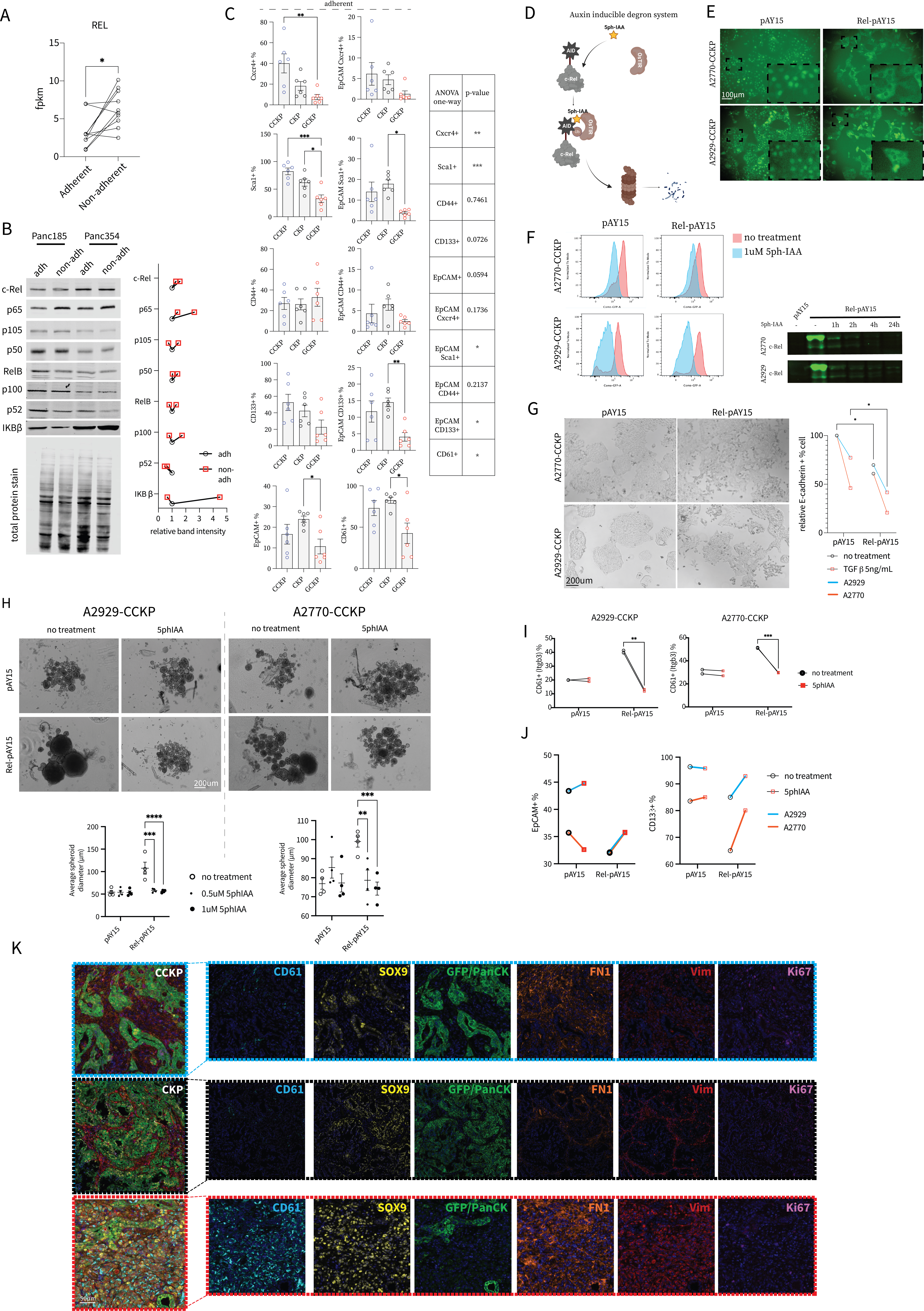

Supplement: Supplementary file 4 — Supplementary Material 4. Supplementary Figure 4. A) REL mRNA fpkm values were compared with paired t tests from an RNA-seq experiment performed with human PDAC cell lines cultured under adherent and nonadherent conditions. B) Western blot analyses of patient cell lines cultured under adherent and nonadherent conditions. The data were normalized to the total protein signal. C) Surface expression of multiple CSC markers was assessed via flow cytometry. For each genotype, 6 separate lines were used. The cells were cultured under standard adherent conditions. One-way ANOVA with multiple comparison test results is given in the figure. D) A schematic demonstrating the auxin-inducible degree 2 (AID2) system. A degron (mAID, miniAID)-tagged c-Rel recruits OsTIR (F74G mutant), which facilitates E3 ligase complex formation in the vicinity after auxin analog (5phIAA) treatment. Subsequent protein ubiquitination leads to proteasome-mediated protein degradation in a short time. E) Green fluorescence images of stably transfected CCKP cells. While the empty vector produced GFP mostly in the nucleus, c-Rel tagged with GFP was present mostly in the cytoplasm. F) Flow cytometry histogram graphs of the GFP signal in degron cells indicating that 5phIAA induced a reduction in the GFP signal after 2 hours. Notably, CCKP cells already have a basal EGFP signal due to the recombined c-Rel flox allele. A time-dependent reduction in GFP-tagged c-Rel expression upon 5phIAA treatment is shown in the immunoblot below. G) Representative microscopy images of CCKP cells stably transfected with either empty vector (pAY15) or c-Rel (Rel-pAY15) and treated with 5 ng/mL TGFβ for two days. The relative surface expression of E-cadherin was analyzed via flow cytometry (c-Rel factor p=0.0078). H) Representative microscopy images of degron cell spheroids. The left and right panels show A2929 and A2770 CCKP cells, respectively, which were stably transfected. To rescue c-Rel expression from high to low, the [file 12943_2025_2486_MOESM4_ESM.png]

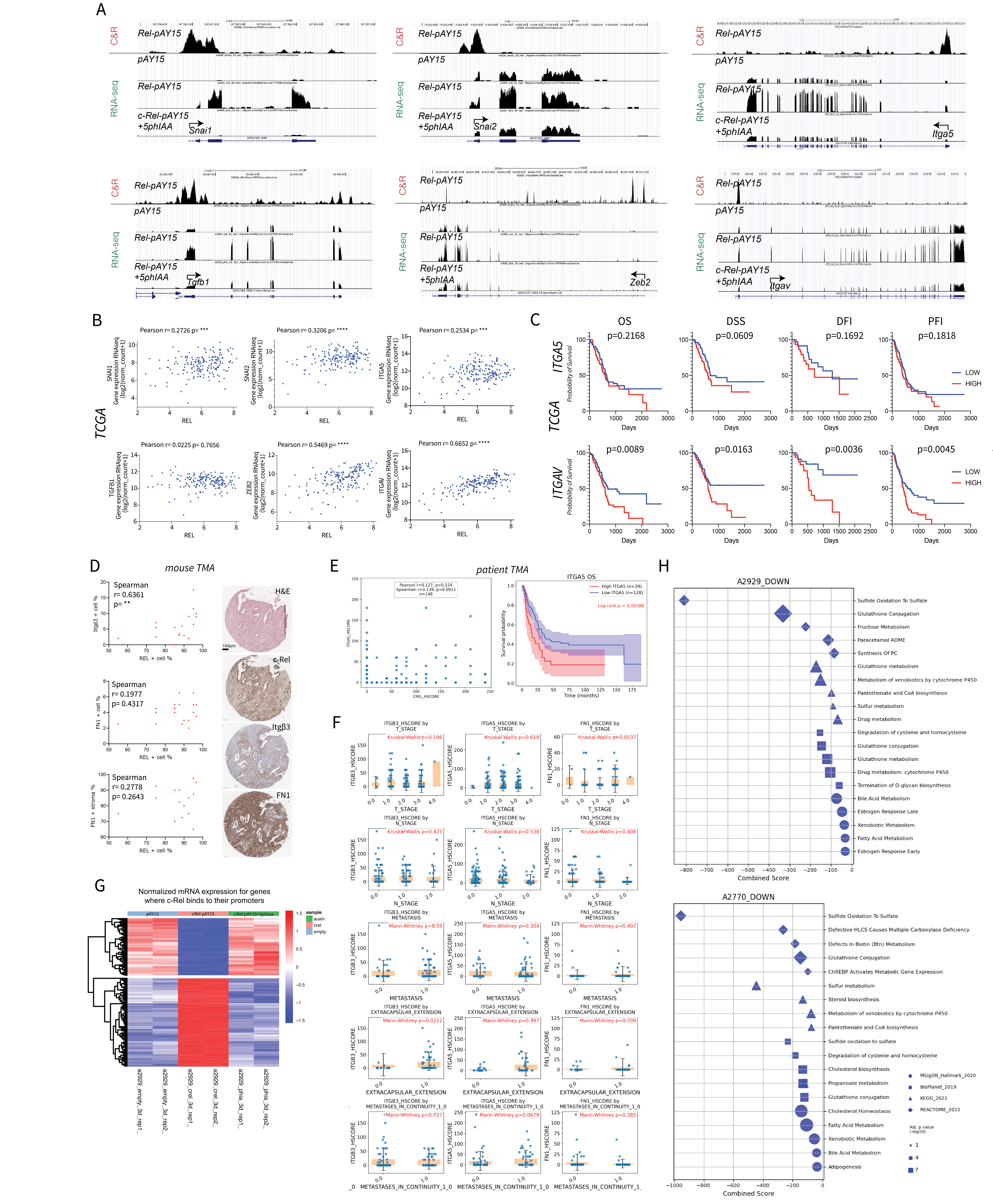

Supplement: Supplementary file 5 — Supplementary Material 5. Supplementary Figure 5. A) CUT&RUN and RNAseq tracks displaying the expression levels of the Snai1, Snai2, Tgfb1, Zeb2, Itga5 and Itgav genes in mock-transfected (pAY15), c-Rel-overexpressing (Rel-pAY15), and degron-induced c-Rel-overexpressing A2929 (Rel-pAY15 + 5phIAA) cell lines. B) Pearson correlations of REL with SNAI1, SNAI2, TGFB1, ZEB2, ITGA% and ITGAV mRNAs in the TCGA cohort. The graphs are adapted from the UCSC-Xena database. C) Kaplan-Meier survival curves for ITGA5 and ITGAV expression in the TCGA cohort. Samples are divided from median expression. OS: overall survival, n= 178; DSS: disease-specific survival, n=172; DFI: disease-free interval, n=69; PFI: progression-free interval, n=178. The graphs are adapted with data from the UCSC-Xena TCGA PAAD dataset. D) Correlation of c-Rel expression with Itgβ3, cancer cell FN1 and stromal FN1 expression in mouse CKP tumors. Representative staining images from the TMAs are given. E) Correlation of c-Rel versus ITGA5 H-scores in patient TMAs on the left plot. On the right, overall survival curve for the ITGA5 H-score given. F) Average patient TMA H-scores are analyzed in samples divided based on various clinical parameters. G) Heatmap displaying DEGs whose promoters are also occupied by c-Rel in degron cells (up- or downregulated). H) Overrepresentation analysis performed by Enrichr for Rel-pAY15 cells. The analysis is performed only with the genes that are differentially expressed and downregulated in Rel-pAY15 cells compared with those in pAY15 only or Rel-pAY15 cells induced with 5phIAA. From each set, only the top 5 hits are selected on the basis of their combined scores. [file 12943_2025_2486_MOESM5_ESM.png]

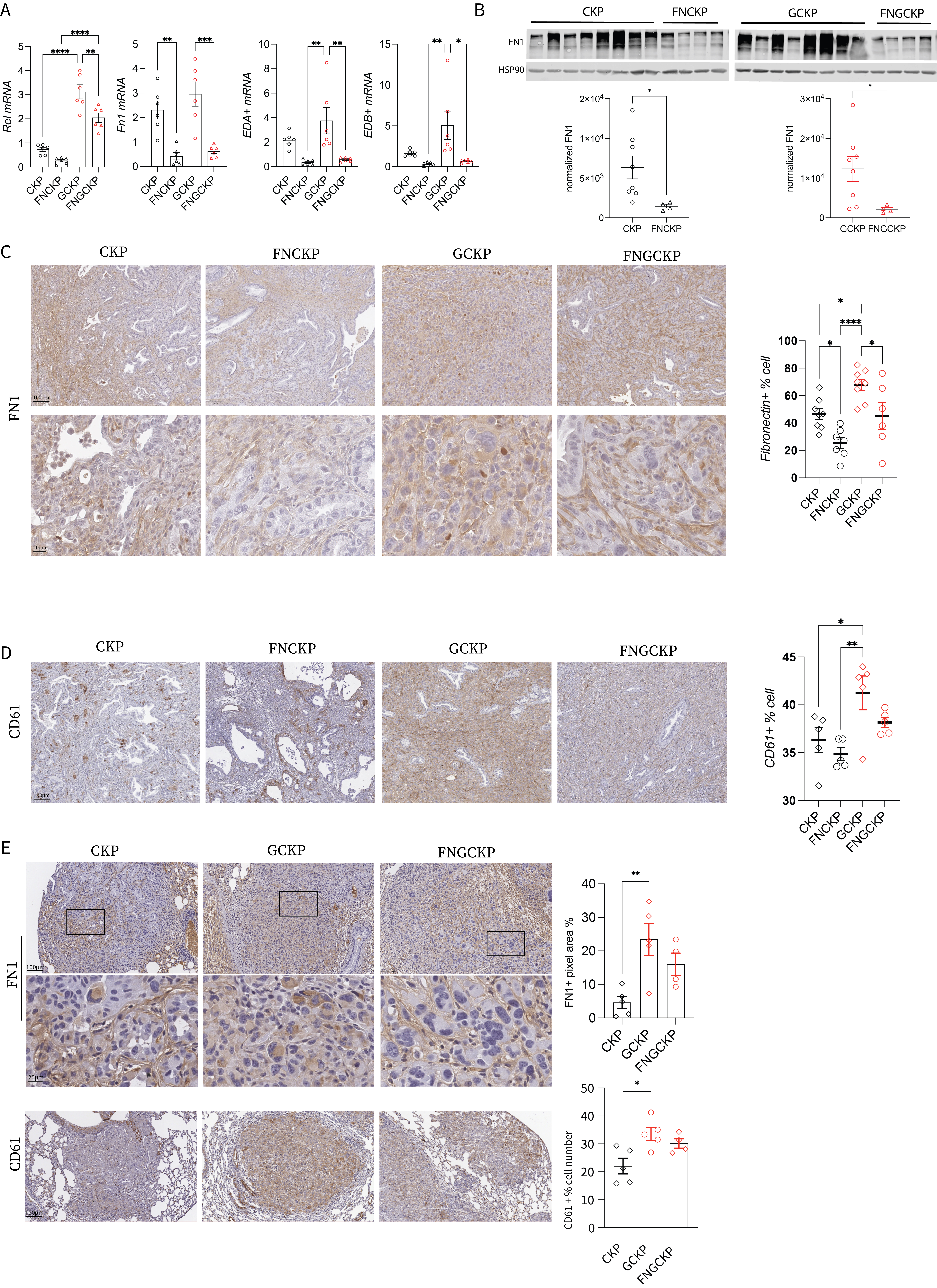

Supplement: Supplementary file 6 — Supplementary Material 6. Supplementary Figure 6. A) RT‒qPCR analysis of the given targets with cDNA synthesized from bulk tumor tissue RNA. One-way ANOVA was used for statistical analyses with p values; Rel p<0.0001, Fn1, p<0.0001, EDA p=0.001, and EDB p=0.0041). B) Bulk tumor tissue lysates were immunoblotted for FN1, and HSP90 was used as a loading control. The relative band intensity quantifications are given below. For statistical analysis, a t test was used. C) Representative FN1 IHC images of the given primary tumor sections and their quantification. The close-up images below indicate the absence of FN1 in cancer cells (One-way ANOVA p=0.0001). D) Representative CD61 IHC images of the given primary tumor sections and their quantification (One-way ANOVA p= 0.0091). E) FN1 and CD61 IHC images of the lung colonies formed after IV injection. (One-way ANOVA, FN1 p= 0.008, CD61 p=0.0144). For all of the one-way ANOVA test graphs, Tukey’s multiple comparison test results are displayed in the figure. [file 12943_2025_2486_MOESM6_ESM.png]
