# Supplementary material for: c-Rel drives pancreatic cancer metastasis through fibronectin-integrin signaling-induced isolation stress resistance and EMT
Source: Mol Cancer. 2025 Dec 15;25:16. doi: 10.1186/s12943-025-02486-5 (PMC12849452; doi:10.1186/s12943-025-02486-5)
Supplement: Supplementary file 7 — Supplementary Material 7. Supplementary Figure 7. Uncropped immunoblot images used in the entire manuscript. [file 12943_2025_2486_MOESM7_ESM.pdf]

Fig 1C

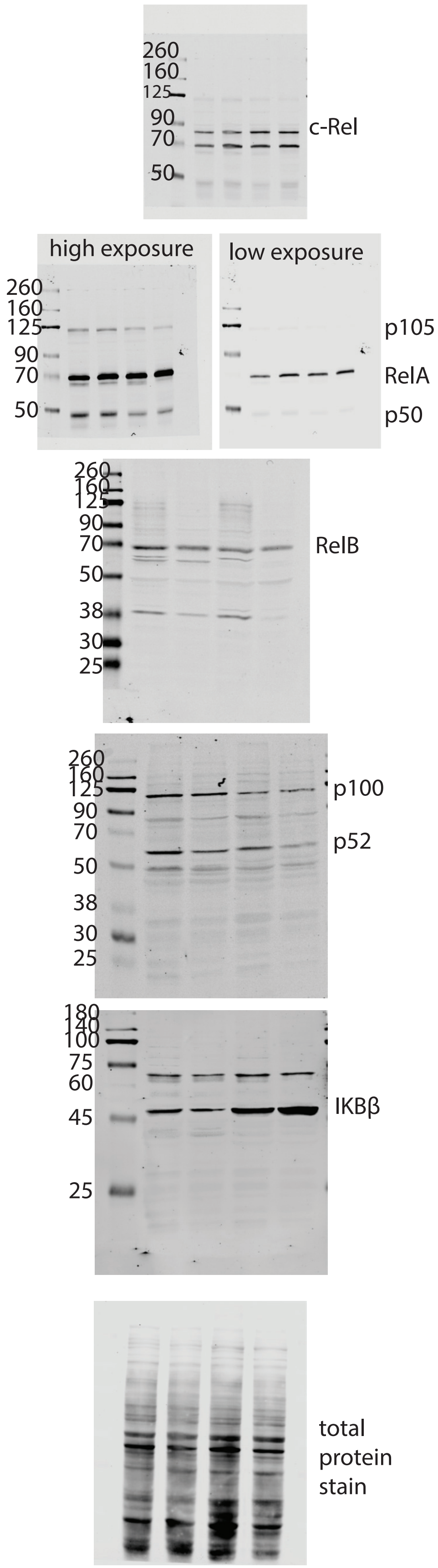

Fig 2B

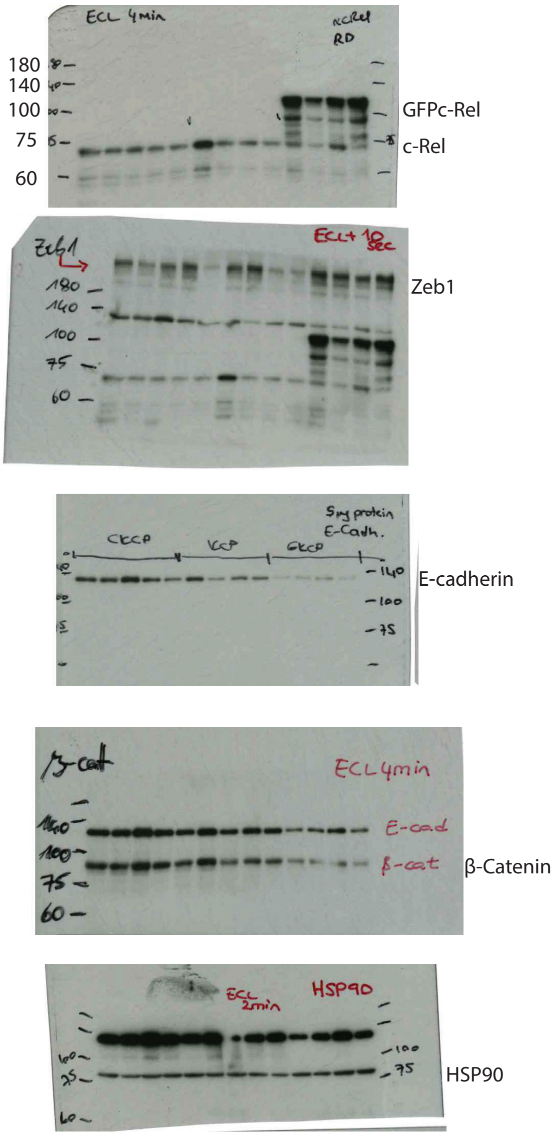

Fig 2E

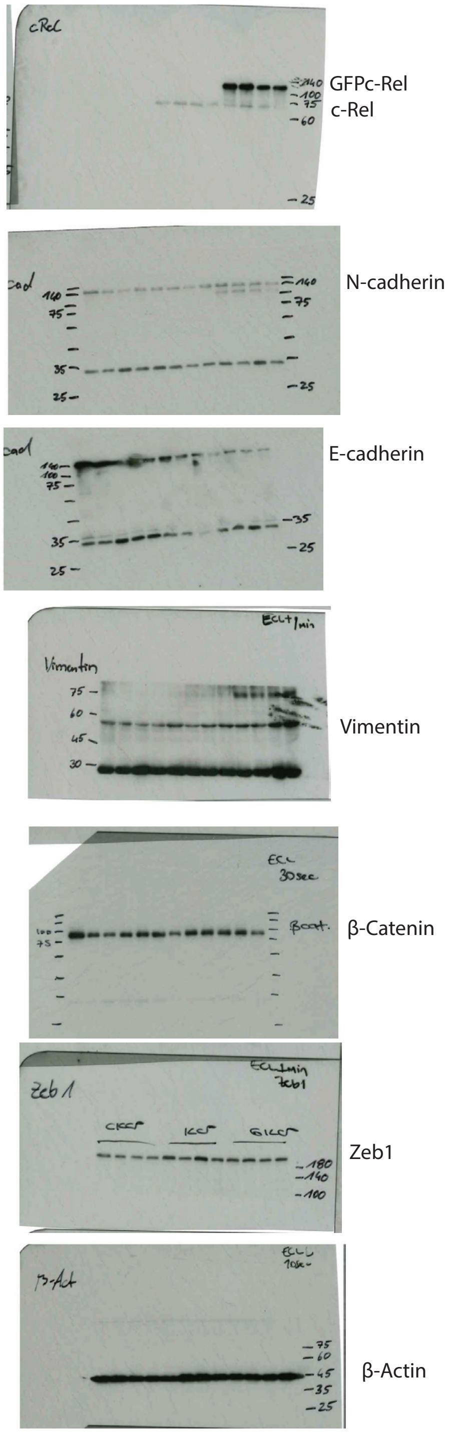

Fig 2I

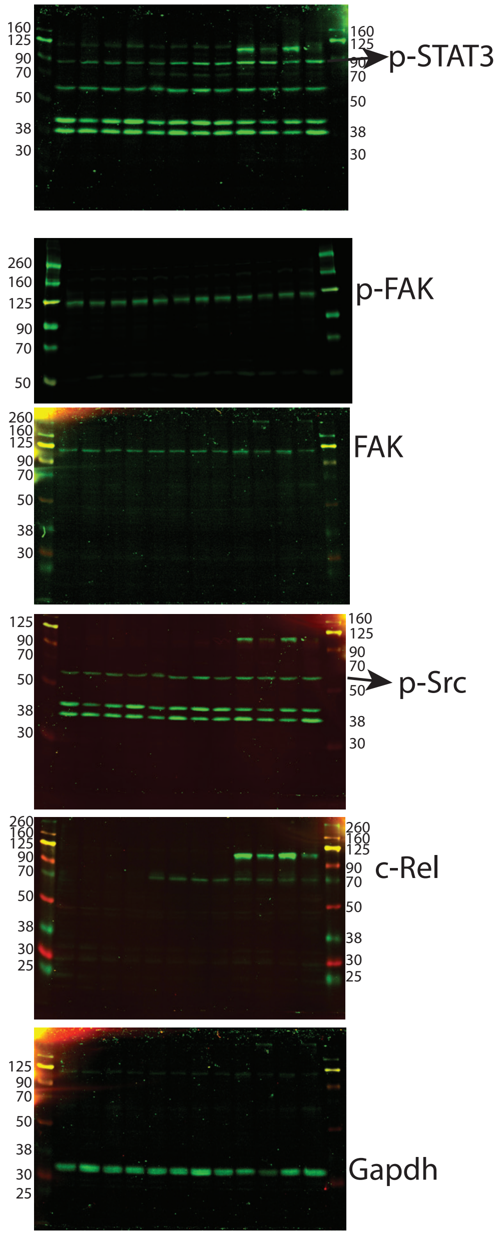

Fig 3C

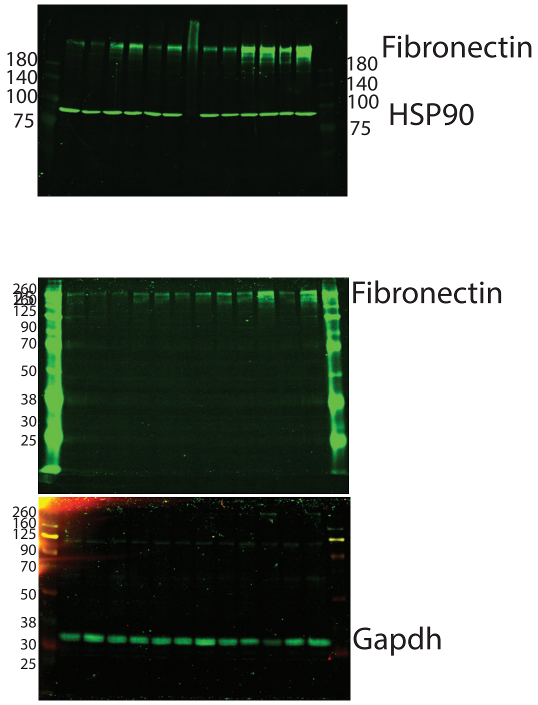

Fig 6F

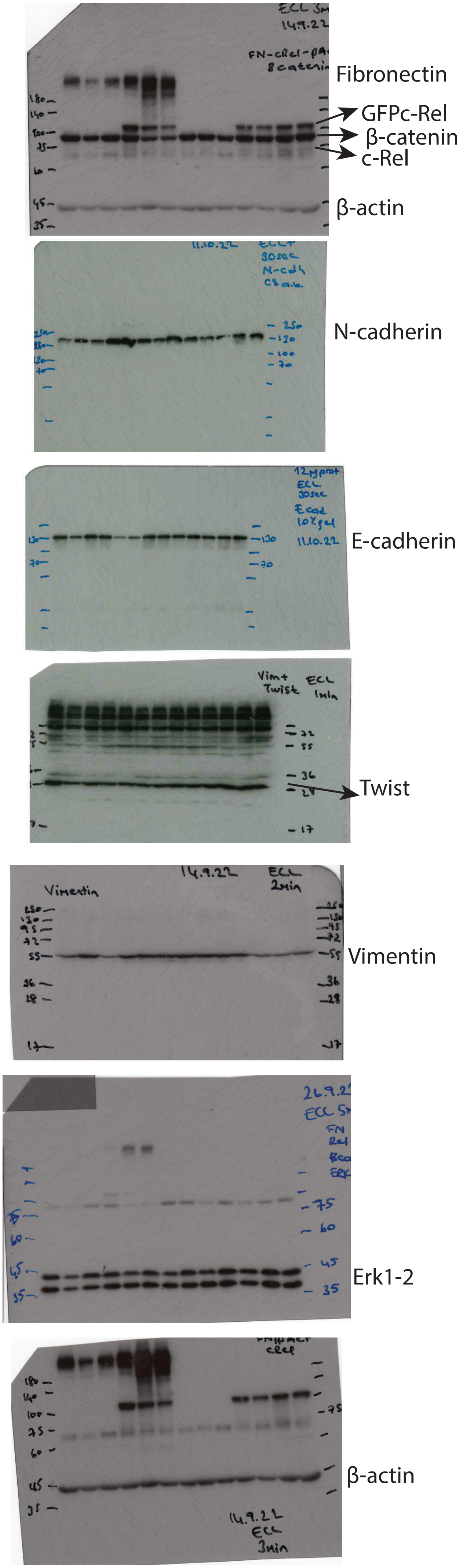

Supp. Fig. 1A

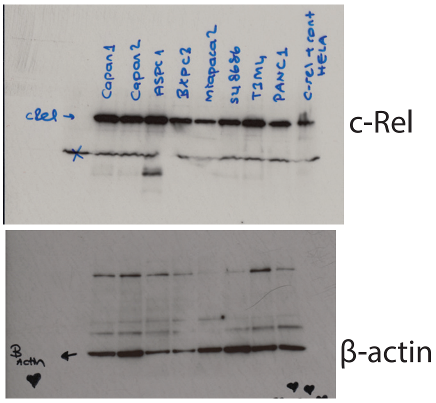

only CK vs CKP used (right 8 samples)

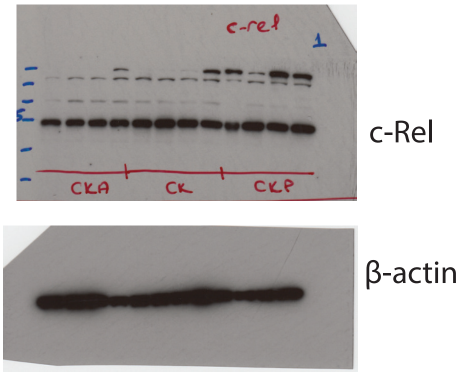

Supp. Fig. 1F

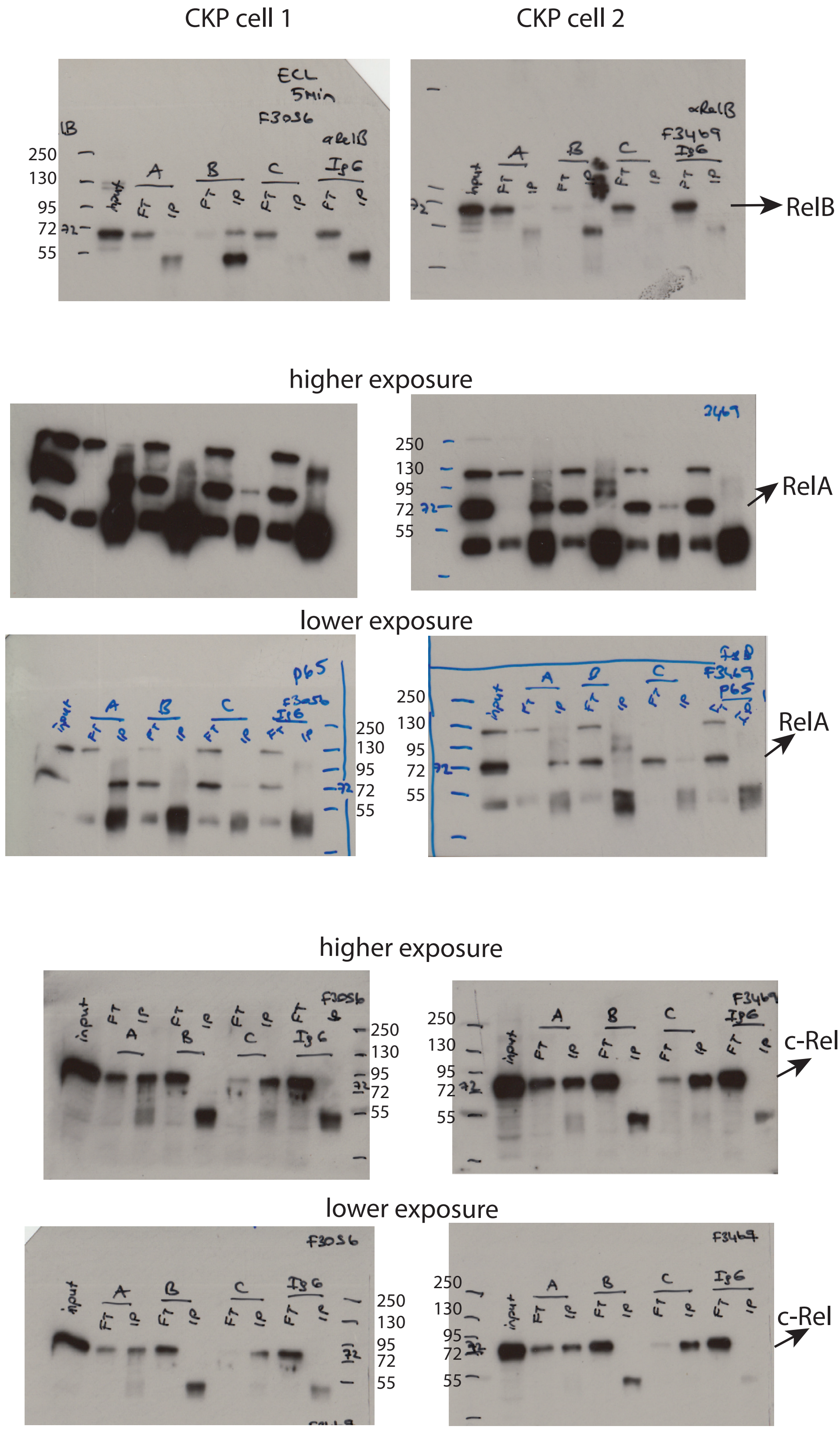

Supp. Fig. 1G

only CKA vs CK used (left 8 samples)

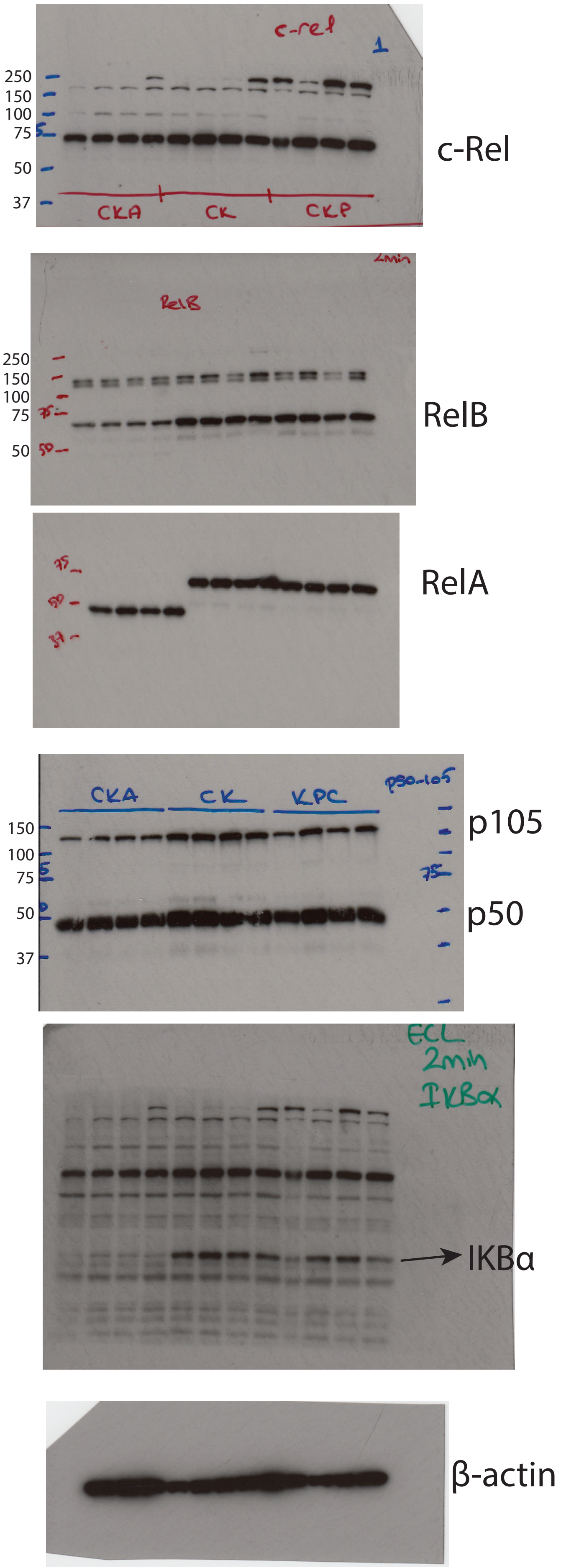

Supp. Fig . 1H

CK vs CKA nuclear extracts

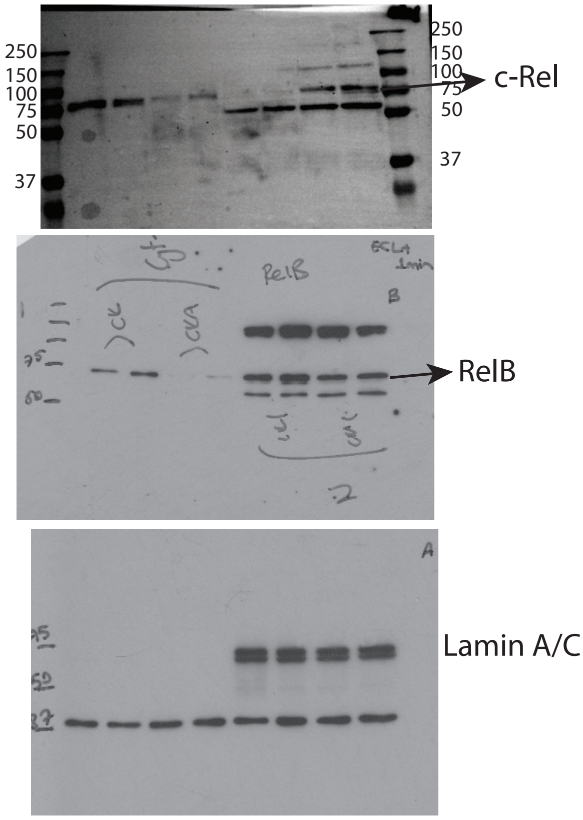

CKP vs CKPA nuclear extracts

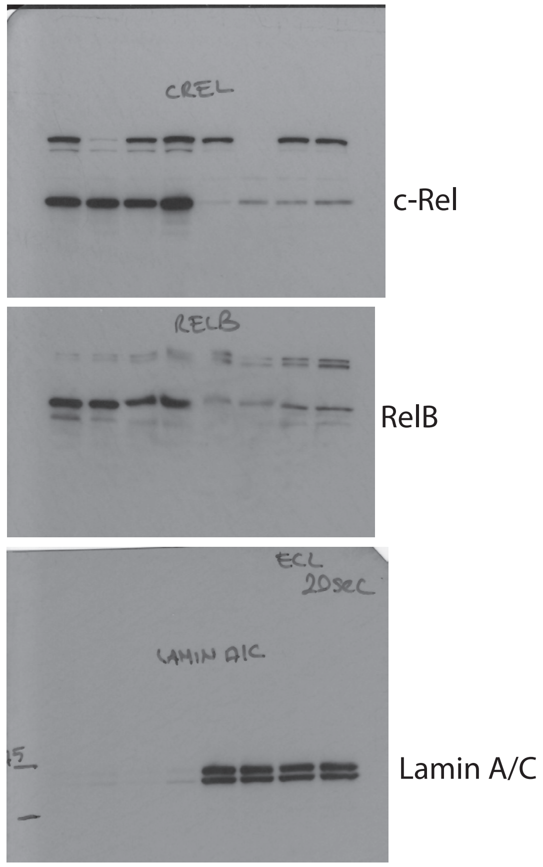

Supp. Fig. 3A

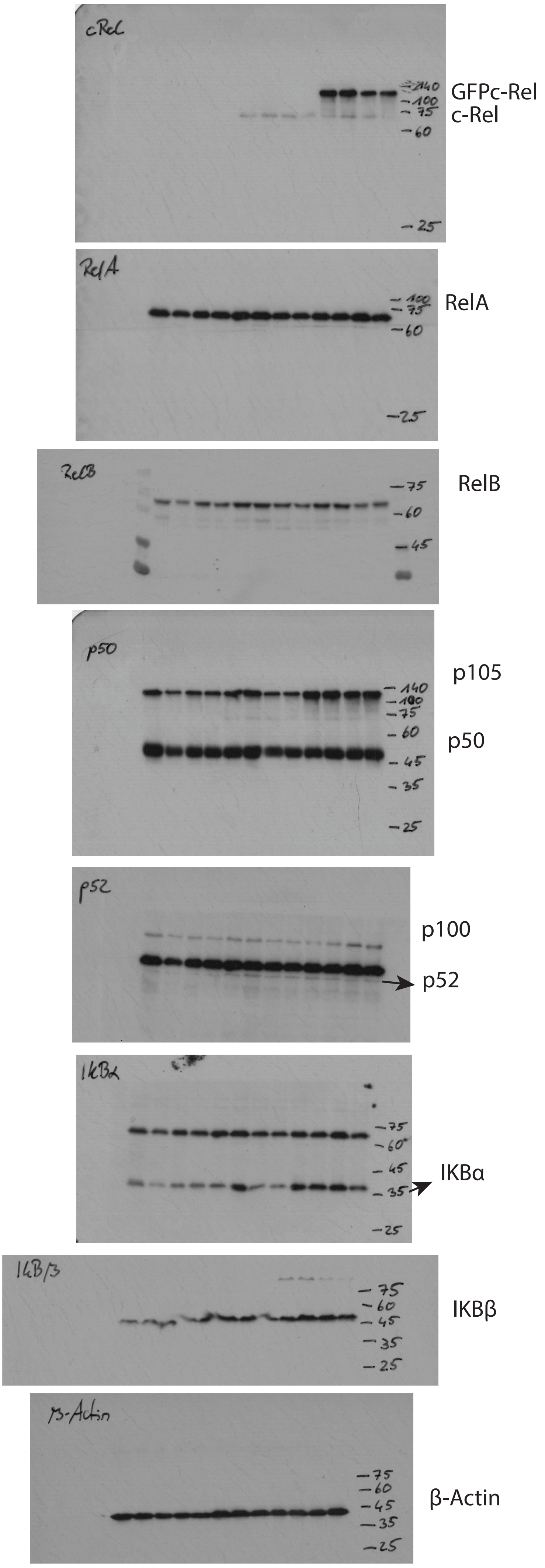

Supp. Fig. 3B

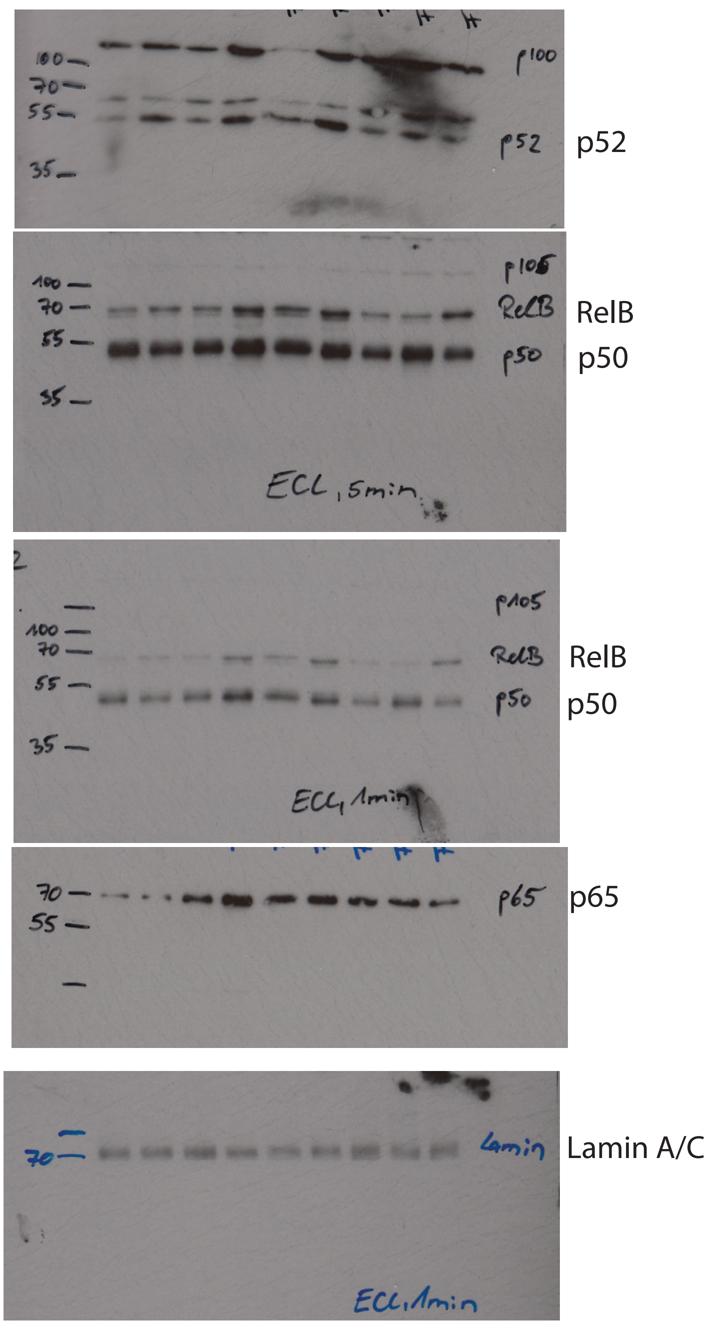

Supp. Fig. 4D

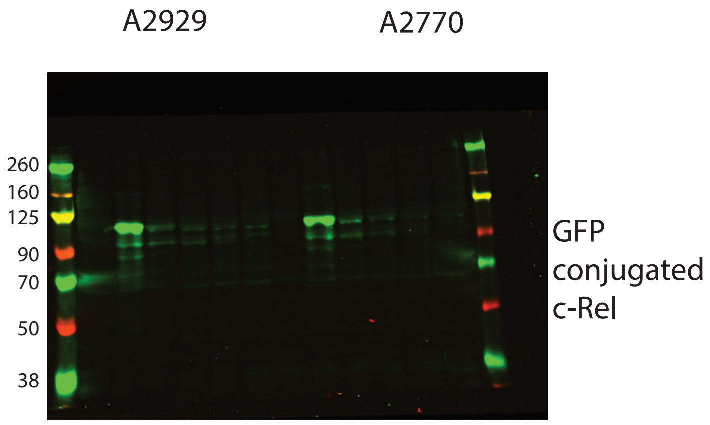

Supp. Fig. 6B

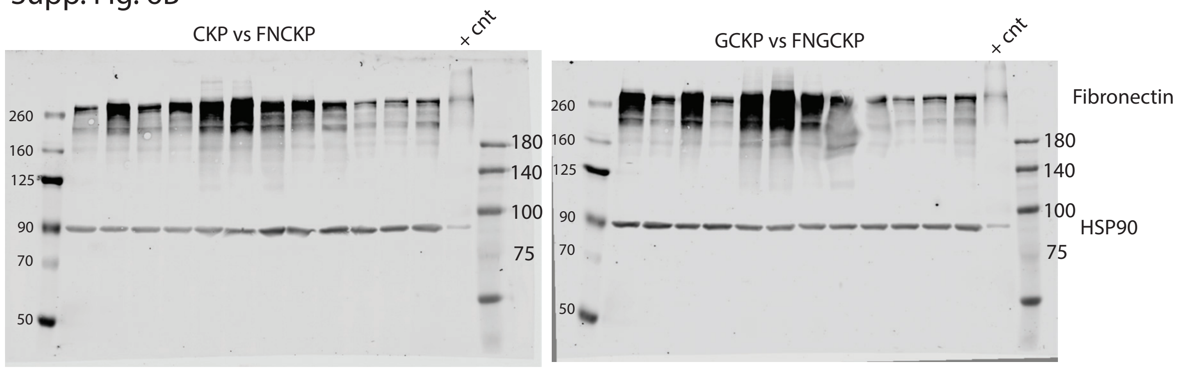

CKP vs FNCKP
+ cnt
260
160
125
90
70
50
180
140
100
75
GCKP vs FNGCKP
+ cnt
260
160
125
90
70
50
180
140
100
75
Fibronectin
HSP90
